# Ontario primary care reform and quality improvement activities: an environmental scan

**DOI:** 10.1186/1472-6963-13-209

**Published:** 2013-06-10

**Authors:** Shannon L Sibbald, Charmaine McPherson, Anita Kothari

**Affiliations:** 1 School of Health Studies, Western University, Labatt Health Sciences Building, Room 222, London, ON N6A 5B9, Canada; 2 Faculty of Information and Media Studies, Western University, London, Canada; 3 School of Nursing, St. Francis Xavier University, Antigonish, Box 5000, Nova Scotia B2G 2W5, Canada

**Keywords:** Quality improvement, Primary healthcare, Human resources, Environmental scan, Policy analysis

## Abstract

**Background:**

Quality improvement is attracting the attention of the primary health care system as a means by which to achieve higher quality patient care. Ontario, Canada has demonstrated leadership in terms of its improvement in healthcare, but the province lacks a structured framework by which it can consistently evaluate its quality improvement initiatives specific to the primary healthcare system. The intent of this research was to complete an environmental scan and capacity map of quality improvement activities being built in and by the primary healthcare sector (QI-PHC) in Ontario as a first step to developing a coordinated and sustainable framework of primary healthcare for the province.

**Methods:**

Data were collected between January and July 2011 in collaboration with an advisory group of stakeholder representatives and quality improvement leaders in primary health care. Twenty participants were interviewed by telephone, followed by review of relevant websites and documents identified in the interviews. Data were systematically examined using Framework Analysis augmented by Prior’s approach to document analysis in an iterative process.

**Results:**

The environmental scan identified many activities (n = 43) designed to strategically build QI-PHC capacity, identify promising QI-PHC practices and outcomes, scale up quality improvement-informed primary healthcare practice changes, and make quality improvement a core organizational strategy in health care delivery, which were grouped into clusters. Cluster 1 was composed of initiatives in the form of on-going programs that deliberately incorporated long-term quality improvement capacity building through province-wide reach. Cluster 2 represented activities that were time-limited (research, pilot, or demonstration projects) with the primary aim of research production. The activities of most primary health care practitioners, managers, stakeholder organizations and researchers involved in this scan demonstrated a shared vision of QI-PHC in Ontario. However, this vision was not necessarily collaboratively developed nor were activities necessarily strategically linked.

**Conclusions:**

Within the scope of this research, the scan affirmed that there is currently no province-wide, integrated, and measured quality improvement program for the primary healthcare sector in Ontario. This could be improved by the development of a coordinated plan, an accompanying accountability framework, and an appropriate sustainable funding envelope for QI-PHC at the provincial level.

## Background

Primary healthcare (PHC) represents the first point of contact between a patient and the healthcare system. PHC is delivered in many settings such as the workplace, home, schools, the family physician’s office, homes for the aged, day-care centres, and community clinics. PHC is also available by telephone, educational health information services, and the Internet [1]. Local delivery of PHC services may exist within a complex public service and non-for-profit “system”, implying that components of the healthcare systems must work together to ensure efficiency and quality. The lack of a national system that identifies governmental roles and responsibilities and that provides a means of communication among local systems could result in a weak infrastructure at the expense of patients. Given its key function as an access point to the rest of the system, the importance of PHC in overall health system strengthening has been affirmed globally [2,3], and there has been substantial reform in PHC by some countries [4-7] as promoted by international agencies such as the World Bank and the World Health Organization [8-10]. There has been much discussion regarding PHC renewal and primary care reform in Canada [10-17]. In particular, the roll out of the Canadian Federal Primary Health Care Transition Fund marked an important shift in focus to PHC [18], allowing various reform strategies, such as quality improvement, to be initiated across the country.

Quality Improvement (QI) is a strategy that aims to improve work processes in order to provide better quality service; although it began in private industry, it is becoming increasingly taken up by the health care system. Management, staff and health professionals work together to increase efficiency and reduce duplication to achieve higher quality patient care. QI needs to be a standard cultural element of PHC practice, rather than a solo activity driven by individual interests to be successful [19]. Evidence has shown that in a variety of settings, dedicated attempts at achieving the attributes associated with QI have directly resulted in a positive trend in patient care and satisfaction [20]. In its simplest form, QI is a formal approach to the analysis of performance followed by systematic efforts to improve it [21]. There are numerous models of QI to support better performance. The definitions of quality and quality-related work often vary depending on the stakeholders involved and encompass a variety of initiatives that have an impact in different contexts. Activities can focus on funding, human resources/expertise, accountability and performance agreements, supporting culture, strategy and structure, or comparative feedback [20].

Focus on QI in health care in North America has increased over the past decade, particularly in PHC in the past five years. These increases can be traced back to key influential events, such as a high impact US Institute of Medicine report on quality in health care [22] and the implementation of the Canadian Federal Primary Health Care Transition Fund [18]. Although individual provinces have increased their focus on QI, there is no guiding framework that supports QI in the PHC sector of the health care system in Canada.

Ontario’s PHC system is publicly funded and offers privately-delivered physician services under provincial funding agreements. Through the past decade, Ontario has developed and implemented a variety of innovative family practice PHC models which have included unique policy incentives (described below). Traditionally, however, most PHC is physician delivered through physician-led clinics; other providers (nurses, social workers, diabetes educators) can also be paid provincially to deliver primary healthcare when the services are rendered through provincial or regional organizations (such as community health centres) or in remote settings. In Ontario, home care and prescription drugs are most often paid for out-of-pocket or by consumer’s private employer-based health plans (exemptions and exceptions apply to special populations such as seniors and veterans).

Ontario has evolved into a national leader in PHC renewal and health system reform with the introduction and growth of Family Health (FH-) Networks, FH-Groups, FH-Teams, FH-Organizations, the expansion of the nurse practitioner role, and the strengthened role of Community Health Centres. The provincial government has invested heavily in response to several important changes in the PHC context including a physician shortage, a growing burden of chronic disease, the rising cost of high quality primary care services, and an overall lack of coordination of care across health sectors [23]. Primary care practices, as a major component of PHC, are organized under different models of care in Ontario to build a more accessible, patient-oriented system and to eliminate the barriers inherent in traditional fee-for-service models [24-27]. During the 1970s, early reform in Ontario introduced Community Health Centres and Health Service Organizations (HSOs). FH-Networks, FH-Groups, FH-Organizations and FH-Teams were established in the early and mid-2000s. As of January 2010, 34% of the Ontario population was enrolled with a FH-Network or FH-Organization (capitation-based models) and 32% was enrolled in a FHG (fee-for-service-based model). Community Health Centres serve 3% of the population [28] while FH-Teams (an interdisciplinary model, most of whose physicians are remunerated through a capitation-based model) serve 16% of Ontarians. There are several notable policy differences among these models, including physician payment schemes, composition and degree of multi-disciplinarity within the team, and priorities, such as populations served and according to which principles. With policy changes in team structure and how care is delivered, the shape and function of inter-professional roles and relationships also shifts [29,30]. (See Additional file 1 for models associated with the QI-PHC activities identified within this scan).

The intent of this research was to complete an environmental scan of QI activities and capacity being built in and by the PHC (QI-PHC) sector in the province of Ontario, Canada. In a complex environment such as PHC, environmental scans are effective tools for gathering diverse data/documentation to help guide policy and decision making [31]. Recent Canadian environmental scans have focused on collaboration of public health and primary care sector [32,33]. As part of the same research program, a similar scan was led by Valaitis et al. [34] to identify and understand examples of PHC and public health care integration in Ontario. It is anticipated that the scan and capacity map presented here will serve as a first step to developing a coordinated and sustainable framework of PHC in Ontario and, given the current worldwide attention to the structure and organization of PHC, as a catalyst for similar frameworks elsewhere.

## Methods

### Context

For the purposes of this study, a broad definition of quality-related work was adopted and included the following aspects: education in quality methods, QI research and program evaluation, performance measurement, quality assessment, quality assurance and accreditation, QI practice facilitation, learning collaboratives, and learning communities.

This work was part of a larger, three-pronged initiative designed to support the development of a coherent, coordinated and efficient strategy for weaving continuous QI into the fabric of PHC in Ontario. This sits within the context of an overall effort to enhance quality across the health care system. The project was done in collaboration with an advisory group consisting of stakeholder representatives and leaders in the delivery and evaluation of QI-PHC in Ontario in order for the results to be relevant for policy and practice. The St. Francis Xavier University Research Ethics Board deemed the project exempt from ethics review given that participants were describing characteristics of programs.

### Data collection

The purpose of the environmental scan was to identify recent, current and planned PHC quality-related activities and capacities in Ontario [35]. After consultation with the advisory group, it was determined that the scan should focus primarily on activities that were either recently completed (i.e., within past year), activities that were currently underway, and activities that would be starting within the next year. The scope of the environmental scan was limited to the following aspects of the quality-related activities: funding, human resources/expertise, tools associated with those activities, and available evidence regarding activity impact.

### Participants and data analysis

A convenience sample, identified by the advisory group, and snowball sampling [36] resulted in 20 key stakeholders in primary health care in Ontario who were interviewed by telephone between January and March 2011; interviews were recorded and transcribed. (See Tables 1 and 2 for participant demographics. See Additional file 2 for interview schedule.) An online review of relevant websites and documents arising from the interviews was then undertaken. Data were analysed independently by two team members (SLS and CM) using rapid Framework Analysis [37] augmented by Prior’s approach to document analysis [38]. Framework Analysis allowed us to explore in our data issues identified by our advisory group (*a priori*) and those that came out of our data (*de novo*). Analysis took place between and among individual activities and the activity pool as a whole. Framework Analysis is a flexible yet systematic method for qualitative analysis that supports the construction of *a priori* thematic categories while allowing other themes to emerge from participant transcripts. Using an iterative process, we identified key themes (or categories) related to the activities arising from the interviews and associated documentary data. We then, as per Framework Analysis methodology, inductively combined and linked data to our *a priori* themes and theoretical propositions of QI-PHC. To integrate Prior’s approach to document analysis, documents were continually re-coded using new themes identified throughout analysis, paying attention to alternate or contrary examples and explanations. To add rigour to our findings, we triangulated by analyzing multiple types of documents (and sources of data). We presented preliminary results to the advisory group both to confirm credibility of findings and to ensure we were considering all relevant issues. Final interpretation drew in known relevant PHC context and background documents.

**Table 1:** Participants by role

**Role (primary affiliation only)**	**Participants**
Researchers	9
Executive/leadership	4
Government officials	2
PHC managers	5
**Total participants**	**20**

**Table 2:** Participants by organization

**Organization category**	**Name of organization**	**Participants**
Government	Ministry of Health and Long Term Care	2
	Cancer Care Ontario	1
	Quality Improvement and Innovation Partnership	1
	Ontario Quality Health Council	1
Professional associations/colleges	Registered Nurses Association of Ontario	1
	Ontario College of Family Physicians	1
	Association of Ontario Health Centres	4
	Association of Family Health Teams of Ontario	1
Research organization	Institute for Clinical Evaluative Sciences	1*
	Élisabeth Bruyère Research Institute	1
Universities	Northern Ontario School of Medicine	1****
	Department of Family Medicine; McMaster University	1
	Quality in Family Practice; McMaster University	1
	Department of Family and Community Medicine; University of Toronto	1
	Departments of Family Medicine & Community Health and Epidemiology; Queen’s University	1
	Centre for Studies in Primary Care, Queen’s University	1*
	Centre for Health Services and Policy Research, Queen’s University	1*
	Department of Family Medicine, University of Ottawa	1**
	Department of Family Medicine, University of Ottawa	1**
	Institute of Population Health, University of Ottawa	1**
	Centre for Studies in Family Medicine, Western University	1***
	**Total organizations represented = 16**	**20**

*Asterisks represent individuals who work in more than one organization. Different participants are represented by the number of asterisks.

## Results

Results are presented in three sections. First we describe the QI-PHC activities/initiatives found throughout our scan. In this section we discuss the groupings (or clusters of themes) discovered through our analysis. Second, we present our ‘capacity map’ for QI initiatives in PHC in Ontario. Finally, we report on key issues identified within the scope of our environmental scan.

### QI-PHC activities and initiatives

Forty-three (n = 43) recent, current, and planned QI-PHC activities were identified by the environmental scan. (See Additional files 3 and 4 for Details related to each activity, including: project title, brief description of project, timeframe for activity, activity leads, funder(s), tools associated with the activity, knowledge translation activities, and contact information). A significant portion of QI-PHC activities (n = 13; approximately 30%) were primarily associated with the Ontario’s six family medicine programs, located within academic health science centres and/or health research institutes (McMaster University, Northern Ontario School of Medicine, Queen’s University, University of Ottawa, University of Toronto and Western University). These programs were often connected with extensive programs of research. Nearly half of QI-PHC activities (n = 21; 49%) were taking place within local and provincial contexts arising from a broad spectrum of the PHC sector. These, and the remaining initiatives, contribute to the overall strength of QI-PHC activities in Ontario.

Analysis of the 43 activities revealed two clusters of quality initiatives.

#### Cluster 1. Long term QI-PHC

This cluster was characterized by a commonality related to time, geographical reach, and programming commitments regarding QI-PHC capacity building. Specifically, the cluster was composed of long-term initiatives in the form of on-going programs that deliberately built in long-term QI capacity building through province-wide reach. *Capacity building* is taken here to mean deliberate efforts to build QI-PHC skills through programming. This is a heterogeneous grouping in terms of governance structures, age of organizations, history of doing QI-PHC work, and other factors.

Cluster 1 activities (n = 22; 51% of all identified activities) were associated with four key province-wide PHC organizations including (listed alphabetically):

(1) The Association of Ontario Health Centres (and its member organizations: Aboriginal Health Access Centres, Community FH-Teams, Community Health Centres), which had the most QI-PHC activities (n = 16). Most activities in this organization focused on developing or implementing QI indicators and tools (n = 6); others were workshops or training based (n = 5), data management based (n = 3), among others.

(2) Cancer Care Ontario had three (n = 3) QI-PHC activities focused on engaging stakeholders (n = 2) and creating a toolbox (n = 1).

(3) The College of Physicians and Surgeons of Ontario had one (n = 1) QI-PHC activity focused on peer assessment as part of a larger quality assurance program.

(4) The Quality Improvement and Innovation Partnership (QIIP) had two (n = 2) QI-PHC activities focused on supporting QI through multiple tools (n = 1) and empirical research in FH-Teams (n = 1).

While the College of Physicians and Surgeons of Ontario is largely member supported, the rest of these organizations are primarily funded by the provincial government. With the exception of The Quality Improvement and Innovation Partnership (QIIP), these organizations have been established for some time, and in the case of The Association of Ontario Health Centres, many of its member Community Health Centres have been in operation for more than 40 years.

The province-wide mandate of these organizations offers a wide and inclusive “reach” and, in some cases, geographically dispersed staff to support the QI-PHC effort. These organizations also have strategic commitments to QI-PHC capacity building operationalized through programming and policy with accompanying accountability frameworks. For example, in Ontario Local Health Integration Networks (LHINs; Ontario’s regional model for coordination of health services) have accountability frameworks where QI is one of the deliverables; community health centres are funded by LHINs and therefore are required to include QI activities. There seems to be a readiness to work across capacity building organizations to support QI-PHC goals as demonstrated by the collaborative work of the organizations (e.g., some Community Health Centres participated in QIIP training).

#### Cluster 2. Limited time QI-PHC

The second cluster (n = 21; 49%) represented activities that were time-limited whose primary aim was research production, including short-term pilots and programs of research. This too is a heterogeneous grouping distinguished from the first cluster by its more local or regional reach. This cluster of activities is diverse and made up of a combination of PHC service models, governance models, and differing commitments to and interests in QI-PHC. Activities in this cluster include full-scale programs of research (n = 8), pilot and demonstration projects (n = 6), training/education programs (n = 5), and committee or collaboration development (n = 2).

The lion’s share of the Ontario Ministry of Health and Long Term Care and national research grant funding for QI-PHC and related activities reviewed is situated primarily within the academic health centres, and are found in this cluster. Out of all 43 activities, 17 (40%) were done either primarily at, or in collaboration with an academic unit; 14 of which were in this cluster (33% of all activities). The largest number of activities were done at McMaster University; n = 7 or 16% of all activities and n = 6 (29%) of all cluster 2 activities). The major strengths of this cluster are: (1) the longstanding leadership and world-renowned PHC researchers within its practitioner-faculty; (2) the university infrastructure that is accustomed to project- and pilot-based funding; and (3) the large, diverse and geographically dispersed population targeted by the activities in this cluster. The informal role of these activities is, fundamentally, to move the QI-PHC agenda through implementation with pilot projects that may, or may not, evolve into sustainable QI programs. Further, the QI-PHC related programs that do exist (e.g., Improved Delivery of Cardiovascular Care Program designed to assisted PHC providers in one region) do not necessarily co-exist within a coordinated QI-PHC plan for the province.

The environmental scan analysis revealed that, in comparison to Cluster 1, this cluster of activities has established research capacity and has attracted funding because of this capacity. However, these activities seem to lack an integrative strategic plan, governance home, and accountability framework that would likely advance a focused and strategic provincial QI-PHC agenda. Further, given the large scope that these activities try to cover within the province, it is remarkable that the majority of these QI-PHC activities rely on a relatively small pool of research leaders and on primarily unpredictable and one-off pilot and project-based funding. Most of this work is done either in-kind or through other grants since the primary care branch at the Ontario Ministry of Health and Long Term Care does not support research and evaluation through its current funding pool.

While Cluster 1 and 2 are unique in scope and duration, they are similar in the fact that there is a significant lack of relative planning between both clusters in terms of an overall province-wide strategy.

### Capacity map for QI-PHC

We collected and analysed data on expertise, personnel, and related funding currently being deployed within the 43 activities in order to better understand Ontario’s capacity for QI-PHC related work. This data informed our capacity map^a^ and represents the human resources component associated with the identified QI-PHC activities. Roles, rather than names, and associated organizational and geographical locations as well as budgetary considerations are provided (see Additional file 5). Significantly, much of the human resource data associated with the reviewed activities was unavailable or was reported as an estimate.

Our analysis identified general capacity patterns for QI-PHC in Ontario. Overall, the pool of existing human resources working province-wide through on-going, long term QI-PHC programs (Cluster 1) is very small relative to the populations and the PHC workforce served by this cluster of activities. QI-PHC expertise for activities under Cluster 1 resides primarily within specific roles within the organization, including a small number of head office staff and regional or outreach staff. The expertise required in these roles included, but was not limited to, advanced program measurement, data management within community-based health services, and QI knowledge capacity building for community-governed PHC services. The Association of Ontario Health Centres for example, had an Education and Capacity Building Team and four Regional Decision Support Specialist positions that worked on the majority of programs. While the Community Health Centre sector has developed internal knowledge capacity with consistent messaging and content to meet QI-PHC strategic priorities, the workload remains largely absorbed within current staffing levels. The situation is similar for Cancer Care Ontario, The College of Physicians and Surgeons of Ontario, and Quality Improvement and Innovation Partnership. In general there is a lack of dedicated human resource capacity in Cluster 1 in terms of QI-PHC programs and PHC workforce.

The capacity for QI-PHC work arising from the second cluster (time-limited) is aligned with the QI-PHC activity trends being primarily project-based and tied to Ontario’s academic health centres. Many of the nationally known leaders in the PHC field come from these geographical areas. They have their own established collaborative networks of investigators, research assistants, and other research personnel to support work. Researchers in these centres are either located within, or well connected to, leading PHC research centres (e.g., Elizabeth Bruyère Research Institute, and the Primary Health Care System Program).

### Key issues in QI-PHC

Six key issues were identified within the scope of this environmental scan that may be interpreted as supports and/or barriers in advancing QI-PHC.

1. Individual commitment

Many PHC clinicians and administrative support staff are dedicated to QI-PHC issues as a part of their everyday work. Interview participants described many impassioned efforts to shift the QI-PHC cultures within their organizations and within provincial stakeholder organizations. This suggests that many stakeholders at the frontline now view QI as a crucial issue, even within their current complex and ever-changing health system environments. Many are fundamentally dedicated to the best possible individual and population health outcomes for Ontarians and their activities suggest that they see QI-PHC as pivotal in the care process. This commitment and readiness are critical supportive factors in advancing the QI-PHC agenda.

2. Work within academic FH-Teams

There is an immense amount of QI-PHC work that has taken place provincially within FH-Teams (n = 10; 23% of activities in this scan), including: committees, large-scale research projects, stakeholder engagement initiatives and educational activities. Many initiatives put forth by universities attempt to integrate cultural change into the academic curriculum of medical students. At the McMaster FH-Team, all staff, from reception to practitioners, have been engaged in completing 66 projects in the last 18 months; these projects, funded by several agencies, address simple logistics of how a clinic runs efficiently to more complex issues of safety in practice.

3. Diverse approaches to QI-PHC

While historically the process of QI in primary care in Ontario, and across Canada, has been limited to professional development in hospitals, the activities in our scan show a shift in this trend through the awareness and attention paid to ‘grass-roots issues. The scope of individual projects varied from individual and physician-centred (for example the Integrating family Medicine and Pharmacy to Advance primary Care Therapeutics (IMPACT) study aimed to improve pharmacist services by integrating services in FH-Teams) to a wider, more regional focus (such as the Improved Delivery of Cardiovascular Care (IDOCC) Program in the Champlain District). Most projects used a multi-disciplinary approach to QI (for example, the Better Innovations Group (“BIG”) Project brought together whole clinical groups in an effort to improve interdisciplinary patient care). The Community Health Centre sector, through many of their QI activities, used a systems-based and patient-centred view of QI-PHC, manifested using workshops, plain-language summaries and stakeholder engagement as part of their QI activities. Systems-wide QI-PHC reform needs to consider these mixed and diverse approaches.

4. Formal governance for QI-PHC capacity

QI-PHC efforts are seriously challenged within the practice setting due to a lack of formal governance system. We found poor tracking of human resources capacity across QI-PHC activities. This was especially true for Cluster 1 activities where QI-PHC human resources capacity (i.e., who is doing what, which portion of an FTE is being used, how much does it cost, etc.) was very difficult to access. To date, leadership has been provided by key organizations and individuals in helping to develop a QI-PHC ‘community of interest’ across the province. This increasing collective interest seems to have had a positive impact on an early stage of integration of activities. PHC practitioners, managers and researchers have been sharing learnings across institutions and, to some extent, practice sectors, and are beginning to work collaboratively on QI-PHC planning and projects. Lessons can also be learned within the sector looking to, for example, The Association of Family Health Teams of Ontario, a newly developed organization with minimal infrastructure and governed by volunteers who are employed full-time in the PHC sector.

5. Knowledge mobilization

There are several knowledge mobilization mechanisms underway that use various strategies (e.g., newsletters, workshops, academic posters, and papers; see Additional files 3 and 4 ‘KM’ column). Most activities had some form of internal and/or external knowledge mobilization most often in the form education/training or workshop (n = 12; 28%) or a report (n = 10; 23%). The majority of activities (n = 26; 61%) had information available on a project specific, or project-linked website. Some knowledge mobilization mechanisms were tied to individual research dissemination efforts, while others were more system-embedded QI programming and expertise development. There were eleven (25%) projects that had no identified knowledge mobilization efforts and three (7%) which, at the time of this scan, had only shared information internally. The lack of website maintenance (updating) related to many identified QI-PHC activities is a barrier if the knowledge is to be shared across stakeholders in a timely manner.

6. Policy directive and leadership

While there has been recent growth in QI-PHC investment by the Ontario Ministry of Health and Long Term Care, the scan findings suggest there is not a Ministry-led coherent QI-PHC policy directive and related plan for the province. Although pilot projects have their place in determining best practices, a string of pilots suggests that the issue of QI-PHC within PHC renewal is not a strategic priority. Despite the fact that our scan identified good programs of QI work and research, it also highlighted a lack of overall coherence and strategy behind the QI-PHC initiatives; at times a duplication of similar QI projects; and definite lack of knowledge mobilization internally and externally.

## Discussion

The PHC system in Ontario is complex and not necessarily interconnected. There are many models of PHC in Ontario each with different strengths and weaknesses [38-40]. The various model contexts, such as the funding mechanisms, human resources and other capacity, and QI accountability expectations, have an impact on the nature of the QI-related activities. There are many opportunities for improvement in client outcomes through enhanced QI processes, for sustainability of the QI changes, and for sharing QI-PHC knowledge across this diverse sector. However, issues related to readiness for continued change, PHC quality cultural shifts, and availability of related sustainable and appropriately targeted funding co-exist within the complexity of health system strengthening through PHC renewal. This diversity needs to be carefully factored into any provincial planning, especially where governance; professional, geographical and organizational jurisdictions; and differing mandates are considered within a provincial PHC plan.

The scan logged many local pilot projects and research activities to support QI-PHC initiatives that are not strategically informing subsequent and broader scale initiatives. The QI activities of PHC practitioners, managers and researchers as well as other PHC stakeholder organizations (e.g., The Ministry of Health and Long-Term Care, Registered Nurses Association of Ontario, Ontario College of Family Physicians, Ontario Medical Association, and Ontario Health Quality Council) involved in this scan demonstrated a shared vision about QI-PHC for Ontario. This vision, however, was not necessarily collaboratively developed and the activities were not necessarily strategically linked.

Accelerating the desired aspects of QI-PHC will likely require increased engagement and leadership from government, professional organizations, and other QI-PHC stakeholders, particularly front-line practitioners and staff. This engagement and leadership must address: (1) QI-PHC human resources/capacity planning; (2) the culture of QI in PHC; (3) system-wide QI-PHC issues including knowledge mobilization.

(1) Build QI-PHC human resources/capacity planning

Capacity for PHC is a challenge in Canada [41] and around the world [42].

Our scan revealed a lack of clarity regarding QI-PHC human resources capacity (i.e., who is doing what, which portion of an FTE is being used, how much does it cost, etc.). In terms of human resources, this workload in Cluster 1 continues to largely be absorbed within current staffing levels. One implication of this current environment for an organization such as Association of Ontario Health Centres is the question of how much of a threshold remains for additional QI-PHC activities on existing staff workloads. Capacity in Cluster 2 was more often aligned with project-based and academic health centres trends; this small pool of PHC researchers is consistent with trends identified by the North American Primary Care Research Group [43], where increasing the number of active PHC researchers is a critical priority for the field.

Although our findings were organized around two different activity clusters, there is also common ground and commitment between the two clusters such that coordination, cooperation and integration across the entire PHC sector may be achievable. The relative planning disconnect between both clusters in terms of QI capacity has obvious implications and limitations. This situation is *incongruent* with other current provincial, national and global environments that have prioritized PHC renewal, and its associated QI-PHC component, within a health system strengthening agenda [44-46].

The implications of the current QI-PHC capacity layout in Ontario is that the capacity appears to be gathered around academic settings for research expertise and research infrastructure and is most often focused on time limited activities (Cluster 2). While QI-PHC capacity seems to have a province-wide spread—sometimes through regions or through individual organizational members - this situation points to the further need for some sort of integrative body that could accurately determine the QI-PHC capacity. Such a system could inform needs and baseline capacity, and track it for system capacity growth and QI intervention outcomes across the province and beyond academic health settings.

The Community Health Centre sector appears to have been developing QI-PHC expertise and processes somewhat separately from the academic FH-Teams and other PHC stakeholders. Much of the work is done ‘in house’ in a collaborative style and without substantial external funding supports. There are likely substantial QI-PHC learnings that would be relevant and could easily be transferred from the Community Health Centre sector to the FH-Teams and other PHC sector and vice versa. Establishing a mechanism (or reconfiguring an existing organization), such as a QI-PHC network or similar community of practice/learning community for Ontario may support knowledge mobilization and thus capacity building. These efforts should be connected to an overall QI-PHC strategic plan and governing body to lead the vision.

Policy development would benefit from continuing with long term capacity building province-wide that is informed by research and local pilot successes. We should also continue to build the evidence base from research, pilots, and other time-limited initiatives to appropriately inform future programming and policy interventions [45].

(2) Improve the culture of QI in PHC

The PHC system is complex, and consequently QI must be flexible and effective in a variety of settings rather than implementation through a prescribed cookbook approach. The current absence of an overarching strategy and direction of PHC activities has been acknowledge by many across Canada [47]. Ontario’s approach has been described as a ‘selection of models’, lacking overarching strategy and direction. [48]. There are many unknowns in terms of how to best advance PHC renewal in Canada [10,49] and in Ontario, in particular. Individual values and behaviour change are integral to a QI cultural shift [50] and have implications for knowledge sharing and collaborative strategy development among the PHC stakeholders [51,52].

There is a vast amount of QI-PHC work that has taken place provincially. In academic health sciences centres, programs that seek to train future healthcare practitioners in the advancement and importance of QI have been laid down in every faculty of medicine at Ontario universities [24], and almost every academic department of family medicine has established QI groups. This demonstrates commitment to advancing QI-PHC, even in the absence of an integrative provincial framework. This commitment is crucial for health care improvements [51].

Given the current state of reform of the PHC system, the opportunity to insert QI as a core activity of reform is ideal. Nevertheless, there are also many unknowns in terms of how to shift the QI culture within PHC service organizations at all levels while also shifting the way that practitioners, managers and funders have traditionally worked [15,28,50]. Thus, efforts to advance QI-PHC should carefully consider that QI is but one aspect of the health system and culture that is being shifted as many other features are concurrently being changed.

(3) System-wide QI-PHC strategy

Ontario lacks a system-wide and sustained approach to QI, and as a result is not equipped to meet the current demands on our PHC such as increased chronic disease, increasing cost of care, and disparate models of care [53]. This creates an opportunity to synergize reform efforts to advance QI-PHC using an integrated and coordinated provincial strategic plan that would include all PHC sectors. There is no integrative Primary Health Care Ontario counterpart to the Association of Ontario Health Centres and Association of Family Health Teams of Ontario to coordinate and to strategically lead a collaborative vision of patient-centred QI-PHC across all PHC sectors. This is clearly a barrier to advancing the QI-PHC agenda [49]. The non-integrated collection of pilots does not constitute a coherent, sustained and strategic program. This presents a significant barrier to QI-PHC that must be addressed if the PHC renewal agenda is to be advanced [49,51].

In recent improvements, QI leadership developers and researchers have begun to create an interconnected network to outline a collective approach to QI in PHC [54]. Going beyond the provincial level, integration on a national level would not only standardize the healthcare system but also increase its rate of improvement by strengthening communication between PHC professionals. One of the ways in which countries around the world have sought to improve leadership and performance is through accreditation [17,55,56]. Pomey and colleagues [57] reported that accreditation to introduce continuous QI programs to newly accredited or not-yet-accredited organizations created new leadership for QI initiatives.

A next step may be to synthesize QI projects so that others (provincially, nationally and internationally) could benefit from the learnings, especially since projects/outcomes might not be published or there may be a lag time in knowledge mobilization [58]. It is important to align provincial funding to meet this need, to target local synthesis and allow PHC stakeholders in the province to mobilize local knowledge across PHC practice settings.

A provincial strategy for advancing QI-PHC should tap into existing knowledge mobilization mechanisms (such as those found in our scan, and notably in the QI-PHC activities of the Community Health Centre sector). Ontario is primed for innovative QI-PHC policy development and sustainability: the internal capacity exists and the opportunity to make meaningful change is feasible through creating a provincial strategy and driving cultural and political change [59].

### Limitations

We acknowledge three major limitations of our study. First, our data collected relied on self-report of our participants, and therefore participants may have been more positive about their projects – however whenever possible we triangulated our findings with websites and/or documentation. Second, in an attempt to gather data that was readily available in a short time frame we may have not captured all programs (current or planned). Similarly, a lack of website maintenance (updating) related to many identified QI-PHC activities might mean that we were missing important information on knowledge mobilization or other activity details (such as human resources). Lastly, and related, using convenience and snowball sampling could be considered a limitation of this study due the potential of not capturing all QI-PHC programs in Ontario during our scan.

## Conclusions

Health system strengthening through PHC renewal is a complex issue. Thus, QI-PHC, as a critical aspect of PHC renewal, is also embedded in these complex health system strengthening and PHC renewal environments.

We should also begin to consider the new priorities and imperatives in quality assessments in designing evaluations of interventions, in developing standards and guidelines for practice (including primary care practice), and in devising approaches to on-going monitoring of the effects of medical practices on the health of patients and populations.

This QI-PHC scan is one piece of the puzzle that can help to provide insight into the complexity of PHC renewal within Ontario’s multidimensional health care system. The scan contributes by identifying a spectrum of recent innovative QI-PHC activities and their related capacities. Sites should be applauded for their efforts to date in improving the quality of care delivered, however there is work to be done to ensure these efforts are not wasted. In this paper we sought out to provide a rich description of these QI activities and capacity being built in and by the primary health care sector in Ontario. In doing this we have also identified six issues that need to be considered by those involved in QI-PHC and by PHC stakeholders more broadly as well as three important strategies for future directions.

Moving towards equitable and accessible health care (fundamental tenets of PHC renewal) will require increased attention to patient-centred QI that flows from consistent leadership and commitment to the issue at all levels and in all PHC locations within the health care system. Health system strengthening though PHC renewal will require a QI-PHC governance system that operates from an integrative accountability framework and towards a common vision. Several key elements required to continue to shift and build a QI-PHC culture and its related capacity appear to exist within Ontario. The supportive elements need to be scaled up for full systems implementation and QI-PHC practice integration.

The potential exists within the 43 identified activities (and likely many that were unidentified) to launch a coherent and collaborative province-wide program of QI-PHC. The challenge is for provincial governmental leadership, in partnership with PHC system leaders, to seize the opportunity to use this potential and effectively advance QI-PHC within the health system strengthening and PHC renewal agenda.

## Consent

Written informed consent was obtained from the participant for publication of this report and any accompanying images.

## Endnotes

^a^The term *capacity map* was predetermined within the terms of this project, so was not changed. This is not to confuse the reader with the *capacity building* language used in describing the Cluster I activities.

## Abbreviations

FH: Family health; PHC: Primary healthcare; QI: Quality improvement; QIIP: Quality improvement and innovation partnership; QI-PHC: Quality improvement activities in primary healthcare

## Competing interests

The authors declare that they have no competing interests.

## Authors’ contributions

AK conceptualized the study, contributed to interpretations of findings, and revised the manuscript for publication. CM contributed to the conceptualization of the study, collected data, interpreted findings and wrote the first draft of the manuscript. SS collected data, interpreted findings, reviewed the literature for the manuscript and finalized the manuscript. All authors read and approved the final manuscript.

## Pre-publication history

The pre-publication history for this paper can be accessed here:

http://www.biomedcentral.com/1472-6963/13/209/prepub

## Supplementary Material

Additional file 1 **PHC Models Covered in Scan.** Describes primary healthcare models associated with the QI-PHC activities identified within this scan. Click here for file

Additional file 2 **Interview Schedule.** Outlines the interview schedule that was followed with participants in order to complete the environmental scan and capacity map. Click here for file

Additional file 3 **Cluster 1: Long term QI-PHC Capacity Building.** Describes long term initiatives for QI-PHC capacity building. Details are provided related to each activity, including: project title, brief description of project, timeframe for activity, activity leads, funder(s), tools associated with the activity, knowledge mobilization activities, and contact information. Click here for file

Additional file 4 **Cluster 2: Time-Limited QI-PHC Activities.** Describes time-limited initiatives for QI-PHC capacity building. Details are provided related to each activity, including: project title, brief description of project, timeframe for activity, activity leads, funder(s), tools associated with the activity, knowledge mobilization activities, and contact information. Click here for file

Additional file 5 **XQI-PHC Capacity Map for Ontario.** presents the current QI-PHC capacity in Ontario identified within the scope of this scan. Click here for file
