# Supplementary material for: Ontario primary care reform and quality improvement activities: an environmental scan
Source: BMC Health Serv Res. 2013 Jun 10;13:209. doi: 10.1186/1472-6963-13-209 (PMC3720221; doi:10.1186/1472-6963-13-209)
Supplement: Additional file 1 — PHC Models Covered in Scan. Describes primary healthcare models associated with the QI-PHC activities identified within this scan. [file 1472-6963-13-209-S1.docx]

**Additional file 1**

**PHC Models Covered in Scan^1^**

|  | **Type** |  |  | **Priorities** |  |  | **Comments** |  |
| --- | --- | --- | --- | --- | --- | --- | --- | --- |
| **Aboriginal**  **Health Access**  **Centres**  **(AHAC)** | |  | Similar to CHCs while offering a blend of traditional Aboriginal approaches to health and wellness and contemporary PHC in a culturally appropriate setting. | |  | 10 AHACs under AOHC  Salaried Model ^2^ | | |
| **Community**  **Family Health**  **Teams (C-FHT)** | |  | Some of the 150 FHTs funded under May 2004 provincial plan to expand access to PHC were funded as C-FHTs, borrowing several of strong features from the CHC model | |  | 20 CFHTs under AOHC  Salaried Model ^2^ | | |
| **Community**  **Health Centres**  **(CHC)** | |  | Designed to meet the needs of a defined community and to provide accessible PHC services to underserved populations within their catchment area; multidisciplinary, prevention & health promotion programs, social determinants of health focus, community governed | |  | 74 CHCs under AOHC (54+ new ones started up in last year or so); Currently one nurse practitioner led team in Sudbury (approx 20 in Ontario now, 19 from other sectors);  Salaried Model ^2^ | | |
| **Family Health Groups (FHG)** | |  | Offer comprehensive PHC services to their enrolled patients; Regular office hours plus extra After Hours blocks of office time and on call to a ministry funded Telephone Health Advisory  Service (THAS); Accessibility focus | |  | 121 in Ontario as of Oct 2009 ^3^  Fee-for-service Model ^2^ | | |
| **Family Health Network (FHN)** | |  | Accessibility, comprehensiveness, doctor-nurse collaboration, use of technology | |  | 33 in Ontario as of Oct 2009 ^3^  Blended Capitation Model ^2^ | | |
| **Family Health Teams (FHT)** | |  | Expected to improve access to PHC for more than 2.5 million Ontarians in 112 communities; Focus on reducing wait times and emergency dept visits | |  | Since April 2005, 150 FHTs have been created in both urban and rural parts of ON; 50 more being planned to bring total to 200 ^4^  Blended Salary Models ^2^ | | |
| **Family Health**  **Organizations**  **(FHO)** | |  | Represents the alignment of Primary Care Networks and Health Service Organizations into one model. FHOs are groups of physicians who provide comprehensive primary health care services to their patients with a focus on illness prevention. | |  | 75 in Ontario as of Oct 2009^3^ | | |

1

Adapted from table presented in Russell, G.M., Dahrouge, S., Hogg, W., Geneau, R., Muldooon, L., & Tuna, M. (2009). Managing chronic disease in Ontario primary care: The impact of organizational

Factors. *Annals of Family Medicine, 7*(4), 309-318

2 Source: Health Force Ontario (2010). *Family Physician Practice Compensation Models.* Retrieved February 15, 2010 from

http://www.healthforceontario.ca/Work/OutsideOntario/PhysiciansOutsideOntario/PractisingInOnt ario/FamilyPhysicianPractice.aspx

3 Source: Province of Ontario (2010b). *Unofficial listing of FHGs FHNs, and PCNs in Ontario as of October 14, 2009*. Ministry of Government Services. Retrieved February 15, 2010 from http://www.onterm.gov.on.ca/ViewRefList_e.asp?list_id=300

4 Source: Ministry of Health and Long-Term Care (2010). *Family health teams*. Retrieved February 15, 2010 from http://www.health.gov.on.ca/transformation/fht/fht_mn.html
