# Supplementary material for: Ontario primary care reform and quality improvement activities: an environmental scan
Source: BMC Health Serv Res. 2013 Jun 10;13:209. doi: 10.1186/1472-6963-13-209 (PMC3720221; doi:10.1186/1472-6963-13-209)
Supplement: Additional file 2 — Interview Schedule. Outlines the interview schedule that was followed with participants in order to complete the environmental scan and capacity map. [file 1472-6963-13-209-S2.docx]

**Additional File 2**

**Interview Schedule**

**************

**Quality Improvement in PHC in Ontario Project**

[Environmental Scan & Capacity Map Piece]

February 2010

- **Purpose:** This project involves completing an environmental scan and capacity map of quality improvement activities and quality improvement capacity that target primary health care in Ontario.
- **Quality-related work** definition includes:

(1) education in quality methods

(2) quality improvement research & program evaluation

(3) performance measurement

(4) quality assessment

(5) quality assurance and accreditation

(6) quality improvement practice facilitation

(7) learning collaboratives

(8) learning communities

- Time parameters: includes recent, current and planned activities. Recent is defined as: since the year 2000 (first year of federal Primary Health Care Transition Fund)

**Opening Questions**

1. Please confirm that you are giving us permission to digitally record our conversation.
2. Please tell me your name and current position.
3. Please confirm that we are permitted to use your name in a listing of interviewees, and to quote you anonymously in any materials related to this project and later related publications. If not, then clarify parameters.

**Identification of PHC Quality Improvement Activities**

1. List the primary healthcare quality improvement activities (QI-PHC) in Ontario that you are aware of.

***Then, take each QI-PHC activity separately and respond to the following:***

1. What was the timing of the activity (actual or proposed start & end dates)
2. Which aspect(s) of the **quality-related work definition** (see above) did the activity fit under (may include a combination of several)?
3. What role, if any, did you play in the activity?
4. Provide a brief overview of the activity and its objectives.
5. Which sector(s) was engaged in this activity?
6. Who was the lead in the activity? Who were other key players?
7. Was this a primarily Ontario-based activity?

If NO, then was it a component of an interprovincial, Pan-Canadian, or international project?

1. What was the funding source(s) for the activity?
2. What were the human resources involved in the activity? Specific sets of expertise? (will help to build capacity map for Ontario, so identify individuals, locations, contact info, specific areas of QI and/or PHC expertise)?
3. Were there quality improvement tools associated with the activity (provide copies, links)?
4. Is there any available evidence regarding the impact of the activity? Impact of the tool?
5. Are there any printed or published materials available regarding the activity (and please forward actual copies, links, contact info, etc.)?

**Closing Questions**

1. Were there key QI-PHC activities in Ontario since 2000 that stand out? Why were they key activities?
2. Are there others involved in QI-PHC activities for Ontario that we should contact?
3. Please add any additional comments that you may have regarding QI-PHC activities and capacity in Ontario.
