# Supplementary material for: Ontario primary care reform and quality improvement activities: an environmental scan
Source: BMC Health Serv Res. 2013 Jun 10;13:209. doi: 10.1186/1472-6963-13-209 (PMC3720221; doi:10.1186/1472-6963-13-209)
Supplement: Additional file 3 — Cluster 1: Long term QI-PHC Capacity Building. Describes long term initiatives for QI-PHC capacity building. Details are provided related to each activity, including: project title, brief description of project, timeframe for activity, activity leads, funder(s), tools associated with the activity, knowledge mobilization activities, and contact information. [file 1472-6963-13-209-S3.docx]

**Additional File 3**

**Cluster 1: Programs for Long Term QI-PHC Capacity Building**

**Association of Ontario Health Centres (AOHC)**

**1. AOHC QI-Related Training**

**2. A Review of the Trends and Benefits of Community Engagement and Local**

**Community Governance in Health Care**

**3. Building Better Teams: Learning from Ontario Community Health Centres**

**4. CHC Logic Model**

**5. Complexity of Care Project Study**

**6. Eastern Region CHC Performance Management Workshop**

**7. Eastern Region Quality Improvement Workshop Spring 2010**

**8. Implementing Dashboards Across CHCs**

**9. Intraprofessional Data Management Committee at Gateway CHC**

**10. Panel Size Study**

**11. Performance Management**

**12. Quality Assurance & Accreditation**

**13. Quality Oversight in Ontario CHCs**

**14. Regional Data Consortium**

**15. Regional Decision Support Specialist Positions**

**16. Supporting New Leaders in Teams**

**Cancer Care Ontario (CCO)**

**17. CCO’s Primary Care Strategy**

**18. IN-SCREEN**

**19.** **Quality in Primary Care - Grand Rounds with Dr. Richard Grol**

**College of Physicians and Surgeons of Ontario (CPSO)**

**20. CPSO Peer Assessment Program**

**Quality Improvement and Innovation Partnership (QIIP)**

**21. QIIP**

**22. Evaluation of QIIP Practice Facilitator Role**

| **1** | **AOHC QI-Related Training** | | | | | | |
| --- | --- | --- | --- | --- | --- | --- | --- |
| Ongoing training (1) Boards in governance, and (2) various workshops regionally and at centres themselves; Large part of training is supporting data management in CHC sector re: data quality; performance management impacting specific deliverables for the professional learning groups | | | | | | |  |
| **Timelines** | | **Leads** | **Funder** | **Tools** | **KM** | **Contact** |  |
| Ongoing for past 5 years | | AOHC Performance Management Committee (PMC) | AOHC | Tools available on website | See website for available materials | Carolyn Poplak  Manager of Education and Capacity Building |  |

| **2** | **A Review of the Trends and Benefits of Community Engagement and Local Community Governance in Health Care** | | | | | | |
| --- | --- | --- | --- | --- | --- | --- | --- |
| This literature review was commissioned by the Association of Ontario Health Centres (AOHC) to gather information and evidence on the concepts of community engagement and community governance within the context of regionalized health systems. The review presents evidence on the positive benefits of citizen engagement and the value added by inclusion of citizens in local organizational community governance in health care planning and decision-making. This literature review looks at citizen engagement and community governance in Ontario as it is believed to have many benefits for health and health care.  The review concludes that enhanced quality of health care, improved individual and community health outcomes, better accountability, and more efficient use of resources are key dimensions of health and health care where engagement of citizens can have a positive impact. | | | | | | |  |
| **Timelines** | | **Leads** | **Funder** | **Tools** | **KM** | **Contact** |  |
| June 2006 | | AOHC  Ktpatzer Consulting | AOHC | N/A | See website for pdf | Carolyn Poplak  Manager of Education and Capacity Building;  ktpatzer@rogers.com |  |

| **3** | **Building Better Teams: Learning from Ontario Community Health Centres** | | | | | | |
| --- | --- | --- | --- | --- | --- | --- | --- |
| AOHC capacity building initiative for Aboriginal Health Access Centres, CHCs, and Community FHTs; The research sought to define, measure and produce recommendations for improving effectiveness in interprofessional teamwork | | | | | | |  |
| **Timelines** | | **Leads** | **Funder** | **Tools** | **KM** | **Contact** |  |
| August 2004 - 2007 | | AOHC in collaboration with University of Toronto, ICES, University of Western Ontario, & Lakehead University | PHCTF;  Health Canada | Tools available on website | 1. Plain language literature review on interprofessional collaboration  2. Five workshops held across Ontario; 6 more workshops held in other regions across Canada (Health Canada funded)  3. Presentations at academic conferences  4. Co-investigators to submit papers to peer reviewed journals; Those published will be posted on AOHC website | [www.aohc.org](http://www.aohc.org) for toolkit  Carolyn Poplak  Manager of Education and Capacity Building |  |

| **4** | **CHC Logic Model** | | | | | | |
| --- | --- | --- | --- | --- | --- | --- | --- |
| CHC Logic Model revision currently underway; will be prepared by June 2010; results-based logic model and evaluation framework for CHC sector; CHC Model of Care on website gives indication of logic model direction; other training and capacity building is based on concepts within the model e.g., community governance, team building, cultural competency | | | | | | |  |
| **Timelines** | | **Leads** | **Funder** | **Tools** | **KM** | **Contact** |  |
| Ongoing since 2007 with recent revision underway | | AOHC Performance Management Committee (PMC) | AOHC | Under development (not yet available) | See website for updates | Carolyn Poplak, Mgr Education and Capacity Building  www.aohc.org |  |

| **5** | **Complexity of Care Project Study** | | | | | | |
| --- | --- | --- | --- | --- | --- | --- | --- |
| Initial pilot of 6 diverse CHCs weighting the client complexity so can compare to other FHTs; measuring who the CHCs see based on co-morbidity data sets from ICES; Aim is to be able to better describe the complex population served by the CHCs and how this relates to complexity and weight of caseloads; Comparing to other primary care provider groups to examine differences and similarities in user population characteristics and thus complexity of care; Supports clinical team accountability through data-driven decision-making at CHC level; Diverse sites included francophone, youth centres, northern, rural and urban; Six CHC pilots finished in March 2010; expanding to provincial analysis of all CHCs this year; regional focus with provincial implementation | | | | | | |  |
| **Timelines** | | **Leads** | **Funder** | **Tools** | **KM** | **Contact** |  |
| Pilot period July 2009-Feb 2010; Initial pilot results presented in Feb 2010; Provincial data collection starts June 2010 and throughout summer, into ICES by Sept 2010 with report by March 31, 2011; ongoing biannual updates & reporting thereafter; possibly reexamine index data every 5 yrs | | AOHC in collaboration with ICES  Co-PIs:  Jennifer Rayner (AOHC)  Rick Glazier (ICES), Co-PIs | Absorbed within Rayner’s role; small contract ($2000) with ICES for initial data storage & database access | Adjusted John Hopkins ACG, and 6-7 databases from ICES utilization bands, including emergency utilization; internal CHC database from ECG | Final report co-authored by Rayner & Glazier not yet released; abstract to be submitted to Data Users conference in Ottawa for Sept 2010 | Contact Jennifer Rayner, London Intercommunity Health Centre 519-660-0874  JRayner@lihc.on.ca |  |

| **6** | **Eastern Region CHC Performance Management Workshop** | | | | | | |
| --- | --- | --- | --- | --- | --- | --- | --- |
| Performance management workshop held in eastern CHC region. Capacity building to support other QI strategic objectives. | | | | | | |  |
| **Timelines** | | **Leads** | **Funder** | **Tools** | **KM** | **Contact** |  |
| Held October 2008 | | RDSS eastern region | AOHC & local supports | None identified | See website for resources | Jamie Maskill, RDSS, eastern region |  |

| **7** | **Eastern Region Quality Improvement Workshop Spring 2010** | | | | | | |
| --- | --- | --- | --- | --- | --- | --- | --- |
| A regional workshop aimed at all levels of staff to showcase QI activities within the region. Peer-reviewed abstracts submitted and reviewed. Keynote speakers not yet confirmed as of march 2010, but aiming to connect well-known QI-PHC experts to the CHC work. | | | | | | |  |
| **Timelines** | | **Leads** | **Funder** | **Tools** | **KM** | **Contact** |  |
| To be held in May 2010 | | RDSS eastern region | Not identified | N/A | Website being developed | Jamie Maskill, RDSS, eastern region |  |

| **8** | **Implementing Dashboards Across CHCs** | | | | | | |
| --- | --- | --- | --- | --- | --- | --- | --- |
| Dashboards being implemented across all CHCs to assist CHC Board of Directors in setting targets that help improve various measures towards better quality care. | | | | | | |  |
| **Timelines** | | **Leads** | **Funder** | **Tools** | **KM** | **Contact** |  |
| Ongoing since 2008 | | AOHC performance management committee | AOHC | None identified | None identified other than internal documents | Contact Jennifer Rayner, London Intercommunity Health Centre 519-660-0874  JRayner@lihc.on.ca |  |

| **9** | **Intraprofessional Data Management Committee at Gateway CHC** | | | | | | |
| --- | --- | --- | --- | --- | --- | --- | --- |
| An interprofessional data management committee was developed that cuts across all levels; Developing standard indicators, etc; Data Management and Quality Committee oversees all of work; Running PDSA data and feeding results back to providers | | | | | | |  |
| **Timelines** | | **Leads** | **Funder** | **Tools** | **KM** | **Contact** |  |
| 2009 - present | | Gateway CHC | Gateway CHC | None identified | None identified | Win Wenton, Executive Director and Laura Cassey, Data management Coordinator |  |

| **10** | **Panel Size Study** | | | | | | |
| --- | --- | --- | --- | --- | --- | --- | --- |
| In Phase I, eastern region contracted with EBRI to examine clinical data at CHCs; Aim was to determine best roster size for NPs and physicians; did not include individual co-morbidity status of clients at time; Phase II to extend the original panel size study connecting it with the Complexity of Care study findings; adding a number of NP teams and case mix into equation; supports clinical team accountability | | | | | | |  |
| **Timelines** | | **Leads** | **Funder** | **Tools** | **KM** | **Contact** |  |
| Ongoing with Complexity of Cars Project; initial reporting end March 2010 | | Simone Dahrouge and Bill Hogg at EBRI were original leads;  Jennifer Rayner to lead this second phase | AOHC; Written into Schedule A of agreement, amount not yet determined;  Partially absorbed within Rayner’s role | Adjusted John Hopkins ACG, and 6-7 databases from ICES utilization bands, including emergency utiilization; internal CHC database from ECG | Initial confidential report to CHC Boards and Executive Directors; not shared publically at this point | Contact Jennifer Rayner, London Intercommunity Health Centre 519-660-0874  JRayner@lihc.on.ca |  |

| **11** | **Performance Management** | | | | | | |
| --- | --- | --- | --- | --- | --- | --- | --- |
| Ontario CHCs’ Performance Management Committee Three Year Plan (2009-2012); Performance Management program focuses on the setting of performance and data standards, sector-wide reporting, decision-support, and development of accountability agreements. 3-yr plan outlines 9 main objectives:  (1) To work with the CHC sector to negotiate accountability agreements with the LHINs that continue to entrench the CHC Model of Care, reduces risks to the boards of directors, identifies accountability indicators that reflect the breadth of the model and ensures multi-year funding with regular annual increases.  (2) To position CHCs in maintaining continuous funding, through developing performance indicators which are feasible and acceptable for implementation in Ontario CHCs and that reflect the full CHC Model of Care.  (3) To improve quality of data by developing and improving tools so that informed decisions can be made at the clinical, centre, regional and provincial level.  (4) To enable CHCs and AHACs to demonstrate the effectiveness of their models of care to improve health outcomes for aboriginal, francophone, racialised and minoritised communities, disabled and other vulnerable populations.  (5) CHCs continue to tell their story in order to increase recognition that CHCs are the effective model of care to improve health outcomes of Ontarians.  (6) To support the Model of Care in CHCs, a full set of indicators that reflects the CHC Model of Care is developed and high quality data is produced that illustrates effectiveness.  (7) To ensure community capacity building is recognised as an essential attribute of the CHC Model of Care, data are collected on at least three Community Initiatives indicators and at least one is an accountability indicator in the next M-SAA for 2011-13.  (8) To demonstrate the comprehensiveness of care and the complexity of clients, a methodology to demonstrate complexity of care for CHCs will be developed and endorsed by CHC Provincial ED Network, MOHLTC and LHINs.  (9) To improve the quality of clinical care, relevancy of programmes, and efficiency and effectiveness of service delivery using timely information produced from good quality data and decision-support tools. | | | | | | |  |
| **Timelines** | | **Leads** | **Funder** | **Tools** | **KM** | **Contact** |  |
| 2009-2012 plan with 2010-2011 deliverables | | AOHC Performance Management Committee (PMC) | AOHC | Workplan and multiple related implementation tools | Internal at this point | Anjoli Misra, Manager, Performance Management, AOHC |  |

| **12** | **Quality Assurance & Accreditation** | | | | | | |
| --- | --- | --- | --- | --- | --- | --- | --- |
| The Building Healthier Organizations (BHO) Accreditation Program of COHI is accessed by AOHC; Performance Management program focuses on the setting of performance and data standards, sector-wide reporting, decision-support, and development of accountability agreements. Accreditation is in collaboration with COHI, some funding to develop; AOHC & COHI collaborate on some initiatives & share membership; modeled after Accreditation Canada; comprehensive website; Accreditation standards and processes are currently being reviewed and revised, focusing in particular on how they could be enhanced to better support organizations in efforts to provide services equitably; measures under consideration include policy/leadership level measures, service accessibility measures and HR measures that impact the promotion of equity. Recommendations currently being solicited from the sector for standards revision. | | | | | | |  |
| **Timelines** | | **Leads** | **Funder** | **Tools** | **KM** | **Contact** |  |
| Ongoing past few years | | COHI  Barbara Wiktorowicz, Executive Director, COHI | AOHC | Embedded within the accreditation program itself | N/A | www.cohi-soci.ca  Barbara Wiktorowicz, Executive Director, COHI |  |

| **13** | **Quality Oversight in Ontario CHCs** | | | | | | |
| --- | --- | --- | --- | --- | --- | --- | --- |
| Project to develop tools to assist CHC Boards with quality oversight | | | | | | |  |
| **Timelines** | | **Leads** | **Funder** | **Tools** | **KM** | **Contact** |  |
| Currently underway | | AOHC PMC in partnership with COHI | AOHC & COHI | None yet identified | Not yet identified | Michael Rachlis and Suzanne Ross  AOHC PMC  ww.aohc.org |  |

| **14** | **Regional Data Consortium** | | | | | | |
| --- | --- | --- | --- | --- | --- | --- | --- |
| South east CHC region; developing indicators and comparing interorganizationally; examining CIHI-PHC indicators; looking at where organizations fall in comparison to others; improving data entry; regional reports to regional executive directors | | | | | | |  |
| **Timelines** | | **Leads** | **Funder** | **Tools** | **KM** | **Contact** |  |
| 2008 - Present | | AOHC-PMC | AOHC | None identified | None identified | RDSS in Southern region – Jamie Maskill  Anjoli Misra, Manager, Performance Management, AOHC |  |

| **15** | **Regional Decision Support Specialist Positions** | | | | | |
| --- | --- | --- | --- | --- | --- | --- |
| Funded by MOHLTC through AOHC; position for each region situated in one CHC administrative home but accountable to all EDs in region; Evaluation of role completed (Lori Zegger); identified gaps with respect to regional-provincial issues; Aim of role is to support evidence-based decision-making | | | | | | |
| **Timelines** | | **Leads** | **Funder** | **Tools** | **KM** | **Contact** |
| Since 2008 | | AOHC-PMC | MOHLTC | Multiple examples on website | Example: "Creating Value with  Information in a Performance  Management Environment" by  Data Management Committee Program Learning Group | For ppt & sample work see http://www.aohc.org/aohc/index.aspx?CategoryID=87&lang=en-CA  Contact Anjoli Misra, Manager, Performance Management, AOHC |

| **16** | **Supporting New Leaders in Teams** | | | | | | |
| --- | --- | --- | --- | --- | --- | --- | --- |
| Ongoing performance improvement package to support QI capacity building for new team leaders. | | | | | | |  |
| **Timelines** | | **Leads** | **Funder** | **Tools** | **KM** | **Contact** |  |
| Ongoing as needed | | AOHC | AOHC | Internal tools available | None identified | Carolyn Poplak  Manager of Education and Capacity Building |  |

| **17** | **Cancer Care Ontario’s Primary Care Program** | | | | | |
| --- | --- | --- | --- | --- | --- | --- |
| The Cancer Care Ontario Primary Care Strategy is a province-wide QI program. It recognizes that family physicians and nurses play a crucial role in cancer care, greatly influencing patients’ participation in cancer screening and providing care and support for patients and their families throughout the cancer journey. To strengthen the connection between family medicine and the cancer system, Cancer Care Ontario created this Primary Care Program in 2008. This program is a key strategy for improving the quality of cancer care in Ontario, as outlined in the *2008-2011 Ontario Cancer Plan*.  **Primary Care and Cancer Engagement Strategy**: To guide its work, the Primary Care Program developed a Primary Care and Cancer Engagement Strategy. This clear plan of action focused initially on improving screening and detection rates within the ColonCancerCheck program and will eventually expand to other screening programs and the whole cancer pathway.  **Provincial Primary Care and Cancer Network:** To implement the Primary Care Strategy across the province, regional primary care leads have been recruited in each Local Health Integration Network (LHIN) to act as local contacts for primary care providers and regional cancer programs in Ontario. Together with the provincial primary care lead, they form a Provincial Primary Care and Cancer Network (PPCCN).  CCO is a case study that has developed QI both in KTE and in measuring for all of Cancer; these processes are extending to Renal diseases and Diabetes.  CCO has developed specialist and PC networks, guidance, implementation strategies, tools, spread, provider reports. | | | | | | |
| **Timeframe** | | **Leads** | **Funder** | **Tools** | **KM** | **Contact** |
| Ongoing since 2008 | | Provincial Primary Care and Cancer Network Management Team:  Dr. Cheryl Levitt, Provincial Primary Care Lead  Dr. Doina Lupea, Program Manager  See website for listing of  Regional Primary Care Leads |  |  | See website for pdfs:   Primary Care and Cancer Strategy brochure   [Journal article - *Canadian Family Physician*, November 2009: Provincial primary care and cancer engagement strategy](http://www.cancercare.on.ca/common/pages/UserFile.aspx?fileId=57968)   [Results of Symposium on the Integration of Family Practices and the Cancer Care System](http://www.cancercare.on.ca/cms/one.aspx?pageId=36698) | http://www.cancercare.on.ca/pcs/primcare/ |

| **18** | **IN-SCREEN (or Integrated Screening)** | | | | | | |
| --- | --- | --- | --- | --- | --- | --- | --- |
| Aim is to improve quality in screening for colorectal cancer. Leadership engagement at regional levels seeking to develop a community of practice/network focused on cancer care in primary care. A system developed at CCO combining a series of different administrative databases (billing, laboratory, results data) around colorectal cancer and FOBT screening. Recently completed pilot project with 110 family doctors, where provided them with administrative data from CCO central depository, and asked them to verify its accuracy. MOHLTC has just funded CCO to also include mammography and cervical screening in integrated manner over next year. Plan to develop systems that help CCO provide individual physician level report to guide screening practices; provide with actual profile of each patient and whether they have been screened or not and aggregate data on how they compare to how they were doing before, and on how they compare to their peers and on how they compare to their LHIN, among other items. Goal is to move to 1,000 family physicians and next year to full 9,000 to cover the province, within administrative data limitations. For March-April 2010: external consulting firm to develop full business plan for the project. Effective knowledge mobilization, focus on priorities, and strict workplan necessary since limited staff time. | | | | | | |  |
| **Timeframe** | | **Leads** | **Funder** | **Tools** | **KM** | **Contact** |  |
| Started in 2007 and is ongoing | | Cancer Care Ontario  Cheryl Levitt lead;  Jill Tinmouth, PI on research side | MOHLTC, portion of $193 million colon cancer sponsorship program, primary care program portion $650,000 annually; CIHR grant application currently under review to extend work | See CCO Toolbox link on website | PHC Summit Jan/10; WONKA; OICR;  ICSQ  Various sessions, see website | www.coloncancercheck.ca  Jill Tinmouth, Clinician Scientist & Assistant Professor, Division of Gastroenterology, Department of  Medicine,Sunnybrook Health Sciences Centre & U of T; Adjunct ICES |  |

| **19** | **Quality in Primary Care - Grand Rounds with Dr. Richard Grol:**  **A Lifetime Involvement in QI** | | | | | | |
| --- | --- | --- | --- | --- | --- | --- | --- |
| A high profile event held on February 4, 2010 "Grand Rounds with Dr. Richard Grol: A Lifetime Involvement in Quality Improvement". Aims were to create an opportunity for knowledge exchange by a larger set of primary care and quality stakeholders from across Ontario and to encourage more understanding of the issues and opportunities for expert input and new partnerships. Dr. Grol is an expert in quality improvement in primary care, having led the European Practice Assessment (EPA) program. The overarching objective for this event was to leverage Dr. Grol's expertise to begin to develop indicators for quality improvement for Primary Care & Cancer, beginning with prevention and screening, and later expanding to the cancer journey. This was a face-to-face meeting held in Toronto and was webcast for remote real time access. | | | | | | |  |
| **Timelines** | | **Leads** | **Funder** | **Tools** | **KM** | **Contact** |  |
| Event held Feb 4, 2010  Grant:  Jan 1 – Dec 31, 2010 | | Collaboration among Primary Care Program of Cancer Care Ontario, McMaster University (Department of Family Medicine), University of Toronto (Department of Family and Community Medicine), Ontario College of Family Physicians, Ontario Medical Association, Ontario Health Quality Council, & Quality Improvement and Innovation Partnership (QIIP).  Cheryl Levitt, provincial primary care lead + steering committee from all co-sponsoring organizations | CIHR: Meetings, Planning & Dissemination Grant: Knowledge Translation |  | 1. Event itself is KM  2. Meeting archived on CCO Primary Care Program website with links on sponsor websites | Cheryl Levitt, MOHLTC, primary care  Doina Lupea |  |

| **20** | **CPSO Peer Assessment Program** | | | | | |
| --- | --- | --- | --- | --- | --- | --- |
| The CPSO Quality Assurance Program develops, establishes and maintains programs and standards of practice to assure the quality of practice of the profession and to promote continuing competence among physicians. Peer Assessment is a CPSO quality assurance program that has been designed to assess and evaluate its members by their own peers—practicing colleagues. The program has been in operation since 1980 and thousands of physicians have been assessed. Each year, most physicians (almost 90%) are found to be practicing in a satisfactory manner and receive useful feedback from their assessor. The program’s emphasis is educational and recognizes and acknowledges the professional’s and CSO’s role our role and responsibility in attaining the best possible patient outcomes. CPSO is committed to developing and maintaining professional competencies and in actively partnering with its members to provide tools and resources, such as the feedback from the Peer Assessment Program.  The 2008-2010 CPSO Strategic Plan focuses on Quality Professionals, Healthy System & Public Trust. This includes Building a Strong Regulatory Foundation as one of its priorities. Under this area, a goal is to significantly increase the number of physician assessments to support the development of a system of continuing professional development and continuing competence. CPSO’s strategic plans noted that they will build the capacity to conduct 2,000 assessments on an annual basis by 2010. A proportion of assessments were tied to identified practice indicators of educational need. | | | | | | |
| **Timelines** | | **Leads** | **Funder** | **Tools** | **KM** | **Contact** |
| Since 1980 | | CPSO Quality Assurance Committee | CPSO |  | Links related to aspects of program on website;  Bulletins from Quality available on website | http://www.cpso.on.ca/members/  peerassessment/ |

| **21** | **Quality Improvement and Innovation Partnership (QIIP)** | | | | | |
| --- | --- | --- | --- | --- | --- | --- |
| The Quality Improvement and Innovation Partnership (QIIP) originated as a project within the MOHLTC. In 2009, QIIP formally incorporated as a non-profit organization and has a funding and accountability agreement with the MOHLTC. QIIP is a provincial organization mandated to build ongoing quality improvement capacity in PHC across the province. As part of its core activity in quality improvement, QIIP works with FHTs, CHCs and other models of primary healthcare to implement multi-session Learning Collaboratives. Expert subject-matter faculty and QIIP’s team of external QI coaches assist the practice teams to learn and apply quality improvement methods including the use of rapid cycle tests of change and performance measurement. Areas of focus for improvement have included chronic disease management (diabetes care), prevention (colorectal cancer screening) and office practice redesign (access and efficiency).To date, QIIP has reached 121 teams primarily involved Ontario’s 150 FHTs, but Community Health Centres and Shared Care Pilot projects have also been reached. Plan is to expand into other practice models through the QIIP Learning Community. The goal of QIIP is to advance the development of a high-performing primary health care system. QIIP’s strategic objectives include:  • To introduce, integrate and spread quality improvement methods  • To build a learning community among primary healthcare practices to share and spread improvements and innovation  • To advance the use of performance measurement to plan, test and evaluate improvements in the organization and delivery of primary healthcare  • To partner with other quality initiatives and programs related to primary healthcare | | | | | | |
| **Timelines** | | **Leads** | **Funder** | **Tools** | **KM** | **Contact** |
| 2007 - Present | | Brenda Fraser:  Executive Director  Nick Kates:  Provincial Lead  Brian Hutchison:  Senior Advisor  + other QIIP staff | 100% MOHLTC  Approx  $6 million/yr; budget negotiated annually | QIIP Learning Community – offering teams a series of action groups to participate in active learning cycles plus the LC gateway (web-based platform) and QI coach support  Multiple tools, resources, events & collaborative opportunities available through website | Numerous;  QIIP Improvement and Innovation Framework  Most recent:  1. Learning  Collaborative  1, 2 and 3 Reports  2. QI Showcase  3. Workshop for new FHTs and Nurse-Practitioner Led Teams, Feb 2-3, 2010  4. Presentation at IHI Conference, Washington, DC March 9, 2010  (Fraser, O’Brien, Kates)  5. ‘Collaborative 3’ Congress completed  May 10, 2010 | Quality Improvement and Innovation Partnership  2345 Argentia Road, Suite 101,  Mississauga, ON  L5N 8K4  905-363-0490  905-363-0491  Email: info@qiip.ca  www.qiip.ca  Brenda Fraser:  brenda.fraser@qiip*.*ca |

| **22** | **Evaluation of QIIP Practice Facilitator Role** | | | | | | |
| --- | --- | --- | --- | --- | --- | --- | --- |
| An Evaluation of Introducing Quality Improvement and Innovation Partnership (QIIP) Practice Facilitators into Family Health Teams and their Role in Facilitating the Objectives of Learning Collaboratives. Worked closely with QIIP Steering Committee to examine the intention and the role of the practice facilitators. Large amount of data collected re: how the facilitators engaged with the learning collaboratives to support their QI work. Examined issues such as how many teams they worked with, how they did this work (from a distance, face-to-face), kinds of activities they conducted, how they used their time, challenges in working with teams. | | | | | | |  |
| **Timeframe** | | **Leads** | **Funder** | **Tools** | **KM** | **Contact** |  |
| 1 year ending summer 2009 | | Rick Birtwhistle Mike Green  Jyoti Kotecha  Grant Russell | MOHLTC competitive research grant $223,400 | Contact project manager for details | Report with MOHLTC; papers underway | Jyoti Kotecha,  Project Manager  kotechj@hdh.kari.net |  |
