# Supplementary material for: Ontario primary care reform and quality improvement activities: an environmental scan
Source: BMC Health Serv Res. 2013 Jun 10;13:209. doi: 10.1186/1472-6963-13-209 (PMC3720221; doi:10.1186/1472-6963-13-209)
Supplement: Additional file 4 — Cluster 2: Time-Limited QI-PHC Activities. Describes time-limited initiatives for QI-PHC capacity building. Details are provided related to each activity, including: project title, brief description of project, timeframe for activity, activity leads, funder(s), tools associated with the activity, knowledge mobilization activities, and contact information. [file 1472-6963-13-209-S4.docx]

Additional File 4

Cluster 2: Time Limited QI-PHC Activities

1. Better Innovations Group (BIG)
2. Cardiovascular Health Awareness Program (CHAP)
3. Centre for HealthCare Quality Improvement (CHQI)
4. Collaborative Mental Health Care Network Mainpro© C Program
5. Celebrate Quality Internationally and in Ontario (CQIO)
6. e-Learning to Enhance Quality Assessment Competencies
7. Group Health Centre
8. Improved Delivery Of Cardiovascular Care (IDOCC)
9. Integrating family Medicine and Pharmacy to Advance primary Care

Therapeutics (IMPACT)

1. Improving Practice Outcomes VIA Electronic Health Records

(IMPROVE)

1. Improvements in Pain Management Project
2. Partnership for Health – A Diabetes Prevention and Management

Demonstration Project

1. Primary and Community Care Committee (PCCC) OMA
2. Quality Improvement Strategic Pillar: University of Toronto,

School of Family Medicine

1. Quality Indicator Project
2. Quality in Family Practice Project
3. Resident First Initiative
4. Violence Reduction Project
5. The Change Foundation Projects
6. Using Computerized Decision Support in Primary Care
7. Web-based Patient Self-Management

| 1 | Better Innovations Group (BIG) | | | | | |
| --- | --- | --- | --- | --- | --- | --- |
| Department of Family Medicine, Queen’s University; Major committee created within the Dept 2-3 years ago in response to the development of the FHT and some initial performance measurement work that suggested that there was readiness to move on accountability, measurement and reporting. The group undertook an extensive series of consultations with the department. Committee make up of whole clinical group, all the allied health professions, physicians, residents—all took several days to develop the team and its workplan. Covers entire team—approx 15 FTEs with 22 – 24 physicians + 68 staff and 50 residents per year. Now integrated into departmental culture; weekly “BIG Briefs” updates before grand rounds, standard item on departmental meeting agendas, quarterly planning meetings, integrating residents’ audit project into BIG planning. Doing a number of projects, e.g. interdisciplinary team functioning proposal to MOHLTC through Health Force Ontario to examine best practices in interdisciplinary team work | | | | | | |
| Timeframe | | Leads | Funder | Tools | KM | Contact |
| 2007 - Present | | Karen Hall Barber | Internal to  Dept | Tools available; different tools for 3-4 working groups | Summary  reports | Karen Hall Barber  kphb@queensu.ca |

| 2 | CHAP | | | | | | | | | |
| --- | --- | --- | --- | --- | --- | --- | --- | --- | --- | --- |
| The Cardiovascular Health Awareness Program (CHAP) is a community-based program that brings together local family physicians, pharmacists, other health professionals, public health representatives, volunteers, and health and social service organizations to work together to promote and actively participate in the prevention and management of heart disease and stroke. Largest Randomized Controlled Trial (RCT) ended in 2006; analyzing data for this now; Blood Pressure (BP) and self-reported cardiovascular factors using ICES administrative data; helping 22 communities 16,000 patients 19 control communities; large initiative involving 250 physicians, 130 pharmacists and 600+ volunteers | | | | | | | | | | |
| Timeframe | | Leads | | Funder | | Tools | KM | | | Contact |
| ongoing since 2000 with several phases | | UBC,  McMaster  University,  EBRI | | Canadian  Stroke  Network;  MOHLTC;  ICES | | multiple teaching aids; see  website  for details | Website publications & newsletters posted up to fall 2009; Main publication available related to the intervention: Carter, M., Karwalajtys, T., Chambers,  L., Kaczorowski, J., Dolovich, L., Gierman, T., Cross, D., Laryea, S. (2009). Implementing a standardized communitybased cardiovascular risk assessment program in 20 | | | www.chapprogram  .ca |
|  | |  | |  | |  | Ontario communities. Health Promotion International | | |  |
|  | |  | |  | |  |  | | |  |
| 3 | CHQI | | | | | | | | | |
| In July 2008 the MOHLTC Ontario Health Performance Initiative chose to join The Change Foundation to create The Centre for HealthCare Quality Improvement (CHQI) at The Change Foundation. Operating arms-length from government, CHQI’s commitment to improving the quality of health care through on-the-ground projects across the province aligns perfectly with the Foundation’s new strategic directions focused on supporting the integration of health services and improving the quality of health services in the community. The partnership shares a focus on accelerating the pace and widening the scope of quality improvement in health care in Ontario. CHQI operates at arms-length from the provincial government. The initiative was established in 2006 to accelerate quality improvement in Ontario to improve system-level outcomes in areas of provincial strategic priority. | | | | | | | | | | |
| Timeframe | | | Leads | | Funder | | | Tools | KM | Contact |
| Ongoing since 2006 with org shift in 2008 | | | MOHLTC with The  Change  Foundation | | MOHLTC at the Change Foundation | | | See website  for avail resources | See website  for avail resources | www.chqi.ca |

| 4 | Collaborative Mental Health Care Network Mainpro© C Program | | | | | |
| --- | --- | --- | --- | --- | --- | --- |
| The Collaborative Mental Health Care Network (CMHCN) program links family physicians from across the province with a General Practitioner (GP) Psychotherapist and Psychiatrist mentor in a collaborative relationship to support easy access to caseby-case support and ongoing continuing professional development regarding mental health care. The program is supported by the MOHLTC. The CMHCN connects family doctor mentees to psychiatrist and GP-Psychotherapist mentors through telephone, email and fax. Mentees may contact their mentors on an informal basis for guidance and support. Formal Continuing Medical Education workshops, small group teleconferences and sessions take place regularly in order to foster group cohesion. These tools help to support and augment the case by case mentoring program. Advice in the areas of diagnosis, psychotherapy and pharmacology is provided to mentees who are matched with mentors based on clinical interests and/or geographic location. | | | | | | |
| Timeframe | | Leads | Funder | Tools | KM | Contact |
| Established  in 2001; now permanent program | | OCFP &  MOHLTC | MOHLTC,  Mental  Health Division grant  initially | Innovative tool  development; see website | National & international recognition for work; Evaluation overview on website | http://dl.dropbox.com/u/49189/  CMHCN1/Collaborative%20  Mental%20Health%20Network  %20Web%20Home.html    Eilyn Rodriguez,  Assistant Executive  Director, Research and  Educational Services,  416-867-9646 Ext: 24 |

| 5 | CQIO: Celebrating Quality Internationally & in Ontario | | | | | |
| --- | --- | --- | --- | --- | --- | --- |
| The McMaster Quality in Family Practice Team organized this knowledge transfer and exchange week in Hamilton, Ontario to Celebrate Quality Internationally and in Ontario (CQIO) in primary care/family practice. Co-sponsored by the McMaster Quality in Family Health Team, CCO, CPSO and OCFP. Quality. The CQIO Week encompassed a broad spectrum of strategic and tactical knowledge exchange meetings throughout March 2 to 6, 2009. It helped to bring many stakeholders together to discuss a provincial QI framework for family practices. A summary of the CQIO Week culminating in the key event—the Quality Initiatives Knowledge Exchange Workshop on March 6—is available as an online webcast, and the full Proceeding are available on this site. As well, documents related to the Practice Manager Workshop are available and a summary of the Workshop is documented in the Proceedings. | | | | | | |
| Timeframe | | Leads | Funder | Tools | KM | Contact |
| March 2-9, 2009 | | McMaster  Quality in  Family  Practice  Team, CCO,  CPSO &  OCFP |  |  | See website for:    CQIO  Proceedings:  download the full Proceedings, as well as presentation slides from the  Conference    Webcast: View the online  webcast of the  CQIO  Conference    Practice  Manager Workshop: download the workshop agenda, a summary of the workshop, and other relevant documents.    CQIO Photo Gallery: view images of the CQIO events | http://www.qualityinfamily  practice.com  /recent-events1/cqioconference-march-2-62009 |

| 6 | e-Learning to Enhance Quality Assessment Competencies | | | | | |
| --- | --- | --- | --- | --- | --- | --- |
| The Ontario College of Family Physicians, under the leadership of Jan Kasperski (Principal) and David Price (co-investigator), received funding for this project that developed the electronic infrastructure and support to enable the effective use of the Quality in Family Medicine tool (see Quality in Family Practice project description). The official project name was: Enhancing Competencies in FHT’s using Quality Improvement as a driver for Learning, Team Building and Innovation.    The goals of this project were to 1) adapt some of the existing outreach presentations/training workshops materials, develop additional training resources on the Quality Assessment Tool and develop them into an Internet-based e-learning program and 2) to facilitate training on how to utilize the Quality in Family Practice program and thus dissemination and uptake of the Quality project objectives, in particular use of the Assessment Tool through the web-based medium.    The project was designed to support 7+1 practices in using the Quality in Family Medicine tool to improve and/or develop effective and efficient structures and organizational and clinical process in Family Health Teams. This program focused on the structure and processes that are needed on an organizational and clinical basis to support all team members in a FHT to work together to provide efficient and effective team-based care. Key members of the Quality Assessment Tool project team worked with McMaster’s Division of e-Learning Innovation to adapt existing workshop materials and resources to create:   - A standalone Web-based interactive presentations that outline some of the key messages that are currently delivered in face-to-face presentations; and - An embedded (or reference-based) ‘help’ resources that can be accessed ‘on demand’ as people are using the Assessment Tool.   The team reviewed and tested the tool, assembled practice tool kits and developed an interactive distance-learning program to support the uptake of the program in family practices. It was anticipated that this program would have the potential to provide the underpinning for the launch a province-wide program to enhance the quality of practices throughout Ontario and Canada. | | | | | | |
| Timeframe | | Leads | Funder | Tools | KM | Contact |
| Jan – Dec 2009 | | Jan Kasperski  (Principal) David Price (coinvestigator), | Ministry of  Health &  Long Term  Care | FHT specific tools on the website. -Web-based educational program to support the use of the tool.  -Feedback on the effectiveness of the  web-based education  vs. facilitator supports for the use of the quality tool.  -Proof of concept with  “new” FHT successfully using the online tool and resources to facilitate development of the FHT and practice. |  | http://www.qualityinfa mily practice.com/aboutquality/e-learningquality-tool |

| 7 | Group Health Centre | | | | | |
| --- | --- | --- | --- | --- | --- | --- |
| Group Health Centre in Sault St. Marie. Serves approximately ½ the population of Sault St. Marie. Strong evidence-based leadership among team that developed and implemented innovation Health Promotion Initiatives (HPI) Program and numerous research projects that have contributed to the Primary Care Excellence Model at the GHC. Developed programs targeting improvements in care for people with congestive heart failure, diabetes, osteoporosis, HIV/AIDS and many other conditions. Recently launched vascular intervention program (VIP). (No individual breakdown of projects available at this time.) | | | | | | |
| Timeframe | | Leads | Funder | Tools | KM | Contact |
| Over past 5 years | | Various team members  depending on  initiative. Strong leadership in past from Dr.  Lee | Various funders depending on project | None  identified | See website | http://www.ghc.on.ca Lewis O’Brien  Obrien_1@ghc.on.ca |

| 8 | IDOCC | | | | | |
| --- | --- | --- | --- | --- | --- | --- |
| The Improved Delivery Of Cardiovascular Care (IDOCC) Program is a voluntary regional program designed to assist primary health care providers in the Champlain District improve the delivery of evidence-based prevention and management strategies for heart disease, stroke and diabetes within their practice.; A five-year Cardiovascular Disease (CVD) Prevention Strategy for the Champlain District (Champlain LHIN serves 1/15th population of ON 1.4 million people; 1,000 family physicians offer primary care services in this LHIN). Out of University of Ottawa, part of research program looking to best understand ways to improve service delivery by physicians through sustained changes in primary care practices. The first phase of the strategy includes the roll-out of six key initiatives to improve CVD prevention in the Champlain District, one of which is the IDOCC Program. The IDOCC Program uses an Outreach Facilitation Model in which skilled health professionals serve as an expert resource to primary care practices. The Outreach Facilitators work with practices to implement evidence-based guidelines for the following risk factors and conditions associated with the prevention and management of coronary artery disease (CAD), Stroke and Diabetes:   - hypertension (blood pressure) - dyslipidemia (cholesterol) - smoking - weight management/ physical activity - management of patients with CAD or peripheral vascular disease (PVD) - management of TIA/stroke - management of diabetes   Outreach Facilitators support practices with:   - organizing work so that prevention and chronic disease management are integrated into routine operation - structuring and implementing specific care improvements identified by your practice | | | | | | |
| - increasing the use of evidence-based guidelines - integrating practice activities with other services, including specialists and community resources | | | | | | |
| Timeframe | | Leads | Funder | Tools | KM | Contact |
| Three phases: 2007-8;  2009-10, 2010-11; by end of Phase 3 will have been rolled out across entire district | | Champlain  Cardiovascular  Disease Prevention Network (CCPN), a collaboration of 15 partner organizations.  Coordinated by the  Élisabeth-Bruyère Research Institute in collaboration with the: University of Ottawa,  Faculty of Medicine;  University of Ottawa  Heart Institute;  Champlain Regional  Stroke Program;  Champlain Local  Health Integration  Network; Pfizer;    William Hogg & Clare Liddy, co-leads + several coinvestigators | MOHLTC  $4 million; Joint funding through grants:  Champlain LHIN; and sponsored in part by Pfizer  Canada  Inc.(a  Founding  Industry Partner of the Champlain CVD  Prevention  Network) | See toolkits on website;  Facilitators use IDOCC Program tools made available to them through the primary care practice, as well as their own resources and tools from other established health organizations. Some tools include:  1) Champlain CVD  Prevention Guideline: provides summary of latest evidence-based guidelines for heart, stroke, and diabetes, as well as key risk factors  (e.g., smoking, hypertension, dyslipidemia) and a comprehensive list of community programs and services.  2) Decision Aid and Risk  Factor Management  Tools: Integrated Risk Factor Screening Tool and Guide for Comprehensive Risk Reduction coupled with the CV Risk Flowsheet | Published economic analysis:  40%  positive return on 1st year of investment Growing body of evidence; accepted  paper in Canadian Family Physician journal;  Presented at CDCCentres for Health Services;  NAPCGR  2009 | whogg@  uottawa.c a T: 613562-4262  ext. 1431    cliddy@s cohs.on.c a T: 613562-4262 ext. 1514    www.idoc  c.ca |

| 9 | IMPACT | | | | | |
| --- | --- | --- | --- | --- | --- | --- |
| Integrating family Medicine and Pharmacy to Advance primary Care Therapeutics (IMPACT) was a large-scale provincial demonstration project supported by the Ontario PHCTF (2004-2006) and is now a MOHLTC funded program. IMPACT aimed to improve drug therapy using a collaborative care model that integrates pharmacists into the primary health care team. The pharmacists' main service was individual patient assessments to identify, prevent or resolve drug-related problems. Quantitative and qualitative methods were used to evaluate the process of integration, pharmacist service uptake, drug-related patient outcomes, and the costs associated with program set up and implementation for sustainability. This multi-site project involved 7 pharmacists, approximately 70 physicians and cover approximately 150,000 patients. Within each practice site, a pharmacist with special clinical training worked 2.5 days per week for 1 year and coordinated a multifaceted intervention aimed at optimizing drug therapy to improve patient outcomes (blood pressure, cholesterol, diabetes, pain control, constipation, etc.) The family physicians and other | | | | | | |
| members of the practice worked closely with the pharmacist in implementing these strategic interventions. Family physicians from a range of practice models (Ontario Family Health Networks, Primary Care Networks, and other types of family physician group practices) participated in this project. Quantitative and qualitative methods were be used to evaluate the process of integration, pharmacist service uptake, drug-related patient outcomes and the costs associated with program implementation for sustainability. The integration of the physicians and pharmacists at the practice sites was evaluated with the aim of generating a practical and transferable practice model. | | | | | | |
| Timeframe | | Leads | Funder | Tools | KM | Contact |
| PHCTF pilot  2004-6; ongoing  MOHLTC program now | | MOHLTC (currently)    Large team of investigator & coinvestgators;  Lisa Dolovich & Kevin  Pottie, McMaster Co-I; Team membership details:    http://www.impactteam.  info/  impactTeam.php | PHCTF $2.5  million 2004-6 and then extended and funded as a regular  program of  MOHLTC | See website for  resources | Main  publication in Clinical  Pharmacy and  Therapeutics; see website for numerous conference abstracts | Lisa Dolovich    www.impactteam.  info |

| 10 | IMPROVE | | | | | |
| --- | --- | --- | --- | --- | --- | --- |
| Improving Practice Outcomes VIA Electronic Health Records (IMPROVE) built on previous work: “Primary health care measures on quality and comprehensiveness – estimation, validation and generalization.” The objectives were to:   1. Retest previously created health administrative (HA) data measures against electronic health record (EHR) measures. This comparison will allow conclusions about the strengths and gaps of HA data for use in province-wide indicators of primary health care (PHC); 2. Add new measures relevant to Ontario’s evolving Family Health Teams that will be tested for validity and generalizability; and, 3. Assess a tool, particularly developed by this project, to provide feedback to family physicians and other PHC providers regarding its impact on improvements in the quality of service provided. This tool can then be used more widely to support and enhance best practices in PHC generally;   Information sheet with bar graphs available | | | | | | |
| Timeframe | | Leads | Funder | Tools | KM | Contact |
| April 2007 - May 2008; the quality  of care outcomes are currently being | | MOHLTC  under funding program Enhancing  Quality in  Primary  Health Care | MOHLTC; ICES | toolkit book in pdf available | Inaugural conference Electronic  Medical  Records  (EMR) in  Primary Care Research: | Moira Stewart: moira@uwo.ca    Doug Manuel:  doug.manuel@ices.on.ca |
| tracked and analyzed | | Moira  Stewart &  Doug  Manuel |  |  | International Perspectives held March 25, 2008 in Toronto; posters with results available in pdf |  |

| 11 | Improvements in Pain Management Project | | | | | |
| --- | --- | --- | --- | --- | --- | --- |
| Project initiated before real QI in PHC work started in Ontario, so not nested explicitly in QIIP; Multidisciplinary team including an occupational therapist (OT) with expertise in pain management, and physicians, pharmacist, social worker; enormous menu of tools: questionnaires to measure outcomes previously developed validated; not available publically yet to be presented in near future | | | | | | |
| Timeframe | | Leads | Funder | Tools | KM | Contact |
| 2007-ongoing  (incl earlier pilot before funded project) | | Dale Gunter,  PI  McMaster FHT | Dept of  Family  Medicine,  McMaster University    $100,000 over 2 yrs | 2 kinds tools - research side and intervention side: SF36  Quality of Life;  Cage D  Questionnaire for etoh and drug use; Kehler-10; + developed own tools around medication use for research side; on intervention side: Materials that participants read, self-care, self-  management, exercise etc | No presentations of pilot work yet | Dale Gunter  McMaster University |

| 12 | | Partnerships for Health –  A Diabetes Prevention and Management Demonstration Project | | | | | | | | | | |
| --- | --- | --- | --- | --- | --- | --- | --- | --- | --- | --- | --- | --- |
| South West LHIN and the South West Community Care Access Centre (CCAC) launched Partnerships for Health – A Diabetes Prevention and Management Demonstration Project; 3 year pilot in south western Ontario launched with the full support and funding from the Ministry | | | | | | | | | | | | |
| of Finance’s *Strengthening Our Partnerships* program, in partnership with MOHLTC. This project represents an $8 million dollar investment into the prevention and management of diabetes and, ultimately, of other chronic diseases. The demonstration project will bring together a wide range of health care partners, including:   - South West CCAC - Brockton Family Health Team - Clinton Family Health Team - Strathroy Medical Clinic - South Bruce-Grey Health Centre, Walkerton Site - Thames Valley Family Practice Research Unit, University of Western Ontario - Huron Perth Healthcare Alliance – Clinton Site - South West Local Health Integration Network There are three key elements to the plan: - Stronger partnerships between family doctors, CCAC case managers and other health care providers to support people with diabetes - Resources to empower and support patients in managing their own diabetes - Information Technology systems to support communication and integration among primary health care providers, specialists, hospitals and patients.   Project will be carefully evaluated and based on final outcomes, could provide a model for other chronic diseases; Will include four distinct phases that clearly identify deliverables and milestones that ensure governance and accountability throughout life of project. Comparing 3 different intervention approaches using facilitators; assesses IT readiness of sites; Care algorithm using Canadian Diabetes Association (CDA), Community Health Representative (CHR) algorithm, workflow processes and standardized forms. Involves more than 50 practices; Many are demonstrating positive results, with patients showing improved A1Cs, blood pressures and blood lipids, and fewer low blood sugar incidents. | | | | | | | | | | | | |
| Timeframe | | | Leads | | Funder | | | Tools | | KM | | Contact |
| 3 yr pilot project launched Feb 2008 –  Jan 2011 | | | Stewart Harris evaluating the  program    Linkage to  South West  LHIN activities is through  South West  LHIN Chronic  Disease Prevention and  Management  (CDPM)  Committee    See Feb/10 list:    http://www.part nershipsforhea  lth.ca/Partners hip_docs/CDP M%20Member%20List%20%20updated% 20 Feb% 2010.  pdf | | Ministry of Finance $8 million over 3 years  (Strengthening  Our  Partnerships  Program) +  MOHLTC | | | See  project website for resources | | See project website for resources | | http://www.partnershipsfor  health.ca/    Annabelle Mackey  South West LHIN  (519) 672-0445, ext. 2573  1 866 294-5446 annabelle.mackey@lhins.o n.ca    Sandra Coleman  Executive Director  South West CCAC 519 641-5496 sandra.coleman@sw.ccacont.ca    Tommasina Conte  South West LHIN  519 672-0445, ext 2566 1 866 294-5446 tommasina.conte@lhins.o n.ca    Mike Hindmarsh hindsighthealthcare@rogers.com |
|  | | |  | |  | | |  | |  | |  |
| 13 | Primary and Community Care Committee (PCCC) | | | | | | | | | | | |
| Joint committee of OMA and MOHLTC. Ministry funded group to improve interprofessional education (IPE) among FHTs. Started in Northeast ON June 2009. Focus on (1) engaging the residents in QI work within the interprofessional team environment and (2) The FHT itself as an IPE environment that requires supportive processes. Multiple IPE activities, such as workshops, related to interprofessional team building. | | | | | | | | | | | | |
| Timelines | | | | Leads | | Funder | Tools | | KM | | Contact | |
| 2009-present | | | | OMA &  MOHLTC    David Topps +  2 other physician  colleagues | | MOHLTC & OMA | Strong qualitative approach to development  initially to lay  groundwork; workshop materials focusing on IPE;  Evaluations built into activities | | Final report related to workshops | | David Topps  David.topps@normed.ca    Northern Ontario School of  Medicine (NOSM), Family  Health Research &  Education Team (FHRET)    Tammy McKinnon tmckinnon@fortwilliamfhn.ca | |

| 14 | Quality Improvement Strategic Pillar: University of Toronto School of Family Medicine | | | | | |
| --- | --- | --- | --- | --- | --- | --- |
| Adopted Quality Improvement and Research & Evaluation as strategic pillars based on internal environmental scan. Will guide activities for next 3 years. Developing a curriculum for Family Medicine that includes quality improvement. Considers the standard for a resident in Family medicine to complete a quality project as part of their residency requirements. | | | | | | |
| Timelines | | Leads | Funder | Tools | KM | Contact |
| 2009 – 2012; planning now with implementation in 2010-2011 academic year | | Lynn  Wilson | Internal planning | Survey developed & available;  Considering IHI.org internetbased modular program | Some poster presentations | www.qualityinfamilypractice.com |

| 15 | Quality Indicator Project | | | | | |
| --- | --- | --- | --- | --- | --- | --- |
| Comprehensive data collection, calculation of quality indicators and feedback process in 7 FHTs in Ottawa and Kingston. Provided teams with detailed custom reports + one hour facilitated feedback session on the FHT performance in different areas. Wanted to examine the challenges in measurement, the possible tools that could be used, and how feedback can be provided to teams. Consent obtained from both the providers and patients to do a fully linked study where providers did questionnaires; practices had their staff fill out a single tool, collecting information about the context of the practice, and then patients did individual pre- and post-visit surveys. Comprehensive chart audit done and included any of their administrative data that was housed at ICES to link at the individual level. Data set for 1000 patients. | | | | | | |
| Timelines | | Leads | Funder | Tools | KM | Contact |
| 2 year project 20079 | | Phase I:  Mike Green & Bill Hogg,  co-leads    Phase II:  Mike Green  & Sharon  Johnson | MOHLTC | Providers tool  completed re: context of practice; Qualitative evaluation interviews | Preliminary findings reported I posters at North  American  Primary  Care  Research  Group  (NAPCRG)  2009; CIHR  Summit  2010; Final report at  MOHLTC;  Drafts of papers underway | Mike Green    mg13@post.queensu.ca |

| 16 | Quality in Family Practice Project | | | | | |
| --- | --- | --- | --- | --- | --- | --- |
| Quality in Family Practice is a project of the Department of Family Medicine at McMaster University. This was designed as a province-wide project similar to the national accreditation programs in Australia, New Zealand and Europe. The vision of this project is that ALL Family Practices in Ontario will provide safe and high quality Primary Care. The mission is to implement a comprehensive and integrated continuous quality improvement program in Ontario that promotes and celebrates excellence in Family Practice. The project is an evidence-based undertaking designed to recommend an interdisciplinary assessment program for family practice in Ontario. Based on extensive research, environmental scan, and information gathered from visiting sites in Australia, New Zealand, and Europe, a set of Quality assessment indicators / tools were developed. They have been piloted (Phase 2), and field tested (Phase 3) with a number of FHTs in Ontario. Since this work was completed in 2008, a Delphi study was initiated to validate and fine-tune the Quality tool / indicators. This process was finalized in June 2009, resulting in a re-write of the indicators and re-grouping them into categories that are better aligned with the Canadian Institute for Health Information (CIHI) and the Health Quality Ontario (OHQC) quality definitions. An updated version of the tool will be available in early 2010. The Quality Project now has four concurrent projects underway: Delphi study, Strategic Planning, Quality in McMaster Family Practice implementation within the FHT Collaborative Initiative, and Quality e-Learning Project. Phase 5: OCFP has taken lead to develop educational template to help with better understanding and management of the indicators. | | | | | | |
| Timeframe | | Leads | Funder | Tools | KM | Contact |
| Ongoing since 2000 (started with 1999 visited by Ronald McFitter) | | McMaster  University, Department of Family Medicine in collaboration  with OCFP    Cheryl Levitt, has been Project  Leader and  PI until recently when taken over by  David Price      Phase V: OCFP taking lead in developing educational template (Anthony Levison &  Linda Hiltz) | Originally was  $250,000 MOHLTC  funding (through  PHCTF); Now $500,000 shared between MOHTC &  OCFP    Phase I: 2003-  2005 PHCTF (with OCFP) Phase II:  2005-2006  MOHLTC  Phase III: 2007-8  MOHLTC    2008-2009: MOHLTC –  Health Force  Ontario    Recent: David Price has received about a $500,000  from MOHLTC to expand the Quality project into the six academic sites and develop capacity in those sites to run an assessment within their own units | Hamilton  Quality  Assessment  tool (see website); Rewritten tool will be available on the web for download + purchase as a book through McMaster  Express  Printing | Extensive library of Quality presentations, publications, newsletters, & other documents on website; Annual reports to MOHLTC;  Canadian  Family Practice journal March  2010 (David  Price);    Quality Book of Tools: Cheryl Levitt lead with coauthor Linda  Hiltz | www.qualityinfamily practice.com |

| 17 | Resident First Initiative | | | | | |
| --- | --- | --- | --- | --- | --- | --- |
| This is one of the Ontario Quality Health Council's responses to the long term (LT) care sector to engage a number of stakeholders in meeting the public reporting piece on quality care in the sector. Developed in response to a ministerial request to report on QI; Focused on QI skill development and Improvements in variety of different clinical areas; 15 improvement facilitators hired with goal of developing a cadre of QI facilitators for LT care sector | | | | | | |
| Timeframe | | Leads | Funder | Tools | KM | Contact |
| Starting in 2010 | | Ben Chan,  OHQC | Multi-year  MOHLTC | advanced access QI Guide for  LT care  Homes Check  website for QI tools in near future | Website not available yet | Ben Chan |

| 18 | Violence Reduction Project | | | | | |
| --- | --- | --- | --- | --- | --- | --- |
| Web-based program being developed by the OCFP in response to recommendations made by an inquest into the workplace death of an RN in Windsor, ON. Comprehensive literature review and collaboration with an architect examining ideal workplace design to minimize violence in the workplace. Will include focus groups with staff re: workplace redesign. Secondarily, will provide a web-based program to help staff identify patients whose behaviour may pose a risk. | | | | | | |
| Timeframe | | Leads | Funder | Tools | KM | Contact |
| Starting in 2010 | | OCFP in  collaboration with Dept of Family Medicine,  McMaster  University + # of other FHTs throughout ON | MOHLTC  Workplace Safety envelope $250,000 +  in-kind from  OCFP | To be determined,  including  web-based  interactive educational programming | forthcoming | OCFP |

| 19 | The Change Foundation Projects | | | | | |
| --- | --- | --- | --- | --- | --- | --- |
| The Change Foundation is an independent health policy think tank that supports health system integration and quality improvement in home and community care in Ontario. The quality improvement research agenda includes initiatives to improve the continuity of care among health-care sectors and to contribute to evidence-based decision-making home/community care, one of several areas of concentration committed to in the Foundation’s strategic plan for 2007-2010. For more on Change Foundation research agenda on home and community-care as of June 2009, see http://www.changefoundation.ca/docs/QIResearchAgenda.pdf | | | | | | |
| Timeframe | | Leads | Funder | Tools | KM | Contact |
| Current & ongoing | | The Change  Foundation | internal | n/a | See website | www.changefoundation.ca |

| 20 | Using Computerized Decision Support in Primary Care | | | | | |
| --- | --- | --- | --- | --- | --- | --- |
| Based on the COMPETE project; computerized decision support tracker integrated into Electronic Medical Records (EMR); COMPETE is the original electronic health research group in Canada and has the largest experience with implementation and evaluation of electronic decision support for patients and providers in the country. COMPETE Ι focused on successful implementation of EMRs in small, community-based primary care offices. COMPETE ΙΙ developed a decision support tool (CII Diabetes Tracker) for the high priority and costly chronic disease, diabetes, then tested it combined with automated telephone reminders, in a large randomized trial.COMPETE ΙΙΙ built on the research initiated in COMPETE I and II. This next stage of investigation was broadened to vascular risk— diabetes, hypertension, cholesterol, previous heart attack or stroke. The electronic health care network was also expanded beyond patients and primary care providers to include specialists and Clinical Care Coordinators. The focus was to optimize patient-clinician interactions with the support of the technology to enhance the quality, safety and efficiency of care. | | | | | | |
| Timeframe | | Leads | Funder | Tools | KM | Contact |
| COMPETE  dates back to 1997; 10 year project  with different phases | | Multiple agencies; Anne Holbrook, PI with 11 coinvestigators (see website  for details) | Multiple  Funders: Health  Canada, Ontario MOHLTC, CIHI; peer-reviewed grant support for each phase | multiple see  website  for details | see website for multiple publications  list accurate  1998-2007 | www.competestudy.com |

| 21 | Web-based Patient Self-Management | | | | | |
| --- | --- | --- | --- | --- | --- | --- |
| Uses a patient-controlled health record module (MYOSCAR) to enhance QI in cardiovascular care; ran pilot with 50 people randomized to use tool or not to determine feasibility, ability to use tool, BP records; overall very happy with tool but no changes in BP recorded | | | | | | |
| Timeframe | | Leads | Funder | Tools | KM | Contact |
| 2008-2009 | | MOHLTC    Lisa Dolovich, co-lead | MOHLTC  $200,000 under Enhancing  Quality in  PHC  (EQPHC) | the  MYOSCAR  program  itself is the tool | no publications yet;  abstracts  NAPCRG;  systematic  review done for the project and overview of pilot work | www.myoscar.org |
