# Supplementary material for: Ontario primary care reform and quality improvement activities: an environmental scan
Source: BMC Health Serv Res. 2013 Jun 10;13:209. doi: 10.1186/1472-6963-13-209 (PMC3720221; doi:10.1186/1472-6963-13-209)
Supplement: Additional file 5 — XQI-PHC Capacity Map for Ontario. presents the current QI-PHC capacity in Ontario identified within the scope of this scan. [file 1472-6963-13-209-S5.docx]

**Additional File 5**

**QI-PHC Capacity Map for Ontario**

| **Project** | **FTE & Role** | **Location** | **Funder** |
| --- | --- | --- | --- |
| **Cluster 1: Programs for Long-Term QI-PHC Capacity Building** | | | |
| 1. AOHC QI-Related Training  Decision Support in Primary  Care | 4.0 FTE AOHC Education and Development team | Toronto | AOHC/MOHLTC |
| 2. A Review of the Trends and Benefits of Community Engagement and Local  Community Governance in  Health Care | # FTEs not identified; written by Ktpatzer  Consulting | AOHC &  Ktpatzer  Consulting in  Toronto | AOHC |
| 3. Building Better Teams:  Learning from Ontario  Community Health Centres | # FTEs not identified;  Collaboration between  Canadian Alliance of  Community Health Centre  Associations (CACHA) and  AOHC; | AOHC Toronto  CACHC  Ottawa | AOHC & CACHA |
| 4. CHC Logic Model | Unidentified portion of AOHC performance management staff roles | Toronto | AOHC |
| 5. Complexity of Care Project  Study | 0.4-0.5 FTE of South West  Region RDSS role | London | AOHC |
| 6. Eastern Region CHC  Performance Management Workshop | Unidentified portion of eastern RDSS position | Ottawa | AOHC In  collaboration with partners |
| 7. Eastern Region Quality  Improvement Workshop Spring 2010 | Unidentified portion of eastern RDSS position | Ottawa | AOHC In  collaboration with partners |
| 8. Implementing Dashboards  Across CHCs | Unidentified portion of AOHC staff & all 4 RDSS roles | Toronto | AOHC |
| 9. Intraprofessional Data  Management Committee at  Gateway CHC | Unidentified portion of  Eastern RDSS role +  Gateway CHC Executive  Director & Data  Management Coordinator roles | Tweed | Gateway CHC |
| 10. Panel Size Study | 0.3 FTE of South Western  Region RDSS role | London | AOHC |
| 11. Performance Management | 1.0 FTE Manager,  Performance Management AOHC | Toronto | AOHC |
| 12. Quality Assurance &  Accreditation | Unidentified portion of  AOHC Manager,  Performance Management  + Manager, Education &  Capacity Building roles | Toronto | AOHC |
| **Project** | **FTE & Role** | **Location** | **Funder** |
| 13. Quality Oversight in  Ontario CHCs | Unidentified portion of AOHC staff roles | Toronto | AOHC |
| 14. Regional Data Consortium | Unidentified portion of  AOHC Manager,  Performance Management  & RDSS roles | Toronto | AOHC |
| 15. Regional Decision Support  Specialist Positions (RDSS) | 4.0 FTEs of RDSS positions (1.0 FTE position vacant at present) | Provincial in 4 CHC regions:  north, east, south & west | Funded by MOHLTC  through AOHC; Amt not identified |
| 16. Supporting New Leaders in Teams | Unidentified portion of  AOHC Education &  Capacity Building Team | Toronto | AOHC |
| 17. CCO’s Primary Care  Strategy | Not identified specifically; 2.0 FTE staff leads in partnership with 13 volunteer regional leads as of April 2010 | CCO in  Toronto with province-wide partnerships | CCO |
| 18. IN-SCREEN (or Integrated  Screening) | Not identified | Not identified | MOHLTC,  portion of $193 million colon cancer sponsor program; primary care program portion $650,000 annually |
| 19. Quality in Primary Care - Grand Rounds with Dr.  Richard Grol: A Lifetime  Involvement in QI | No assigned FTEs | webcast | Hosted by cancer Care Ontario |
| 20.CPSO Peer Assessment  Program | Not reported | CPSO in  Toronto with peer assessors province-wide | CPSO |
| 21. Quality Improvement and  Innovation Partnership (QIIP)  Learning Collaboratives and  Learning Community | 1.0 Director, QI  1.0 Manger, QI Initiatives  and  Coaching  16.0 FTE QI Coaches  1.0 FTE Co-leads QI and  Clinical Integration | Active learning cycles/learning sessions and regionally based support to PHC teams across the province | MOHLTC |
| 22. Evaluation of QIIP  Practice Facilitator Role | 1.0 FTE Project Manager + unidentified FTEs for Research support staff | Kingston  (Queen’s  University) | MOHLTC  $223,400 |
| **Project** | **FTE & Role** | **Location** | **Funder** |
| **Cluster 2: Time-Limited QI-PHC Activities** | | | |
| 1. Better Innovations Group  (BIG) | unidentified | Kingston  (Queen’s) | Internal, Dept of Family Medicine |
| 2. CHAP | 4.0 FTE researchers; 10.0 FTE in communities for program delivery | Hamilton  (McMaster) &  Ottawa (EBRI) | Not identified |
| 3. CHQI | No FTEs identified; roles include:  1 executive Director  1 Senior QI Consultant  1 Office Manager  ! Admin Assistant  12 QI Consultants | Toronto | MOHLTC;  The Change  Foundation |
| 4. Collaborative Mental  Health Care Network  Mainpro© C Program | Now absorbed within OCFP  staffing roles | Toronto (OCFP) | MOHLTC,  Mental Health Division grant initially; program of OCFP now |
| 5. CQIO | Not reported; short term event | n/a | Not reported |
| 6. e-Learning to Enhance  Quality Assessment  Competencies | Unidentified (under Quality in Family Practice project— see # 18) | Hamilton (McMaster) | unidentified |
| 7. Group Health Centre | No projects in particular detailed; capacity within center staff for multiple QI projects | Sault St. Marie | n/a |
| 8. IDOCC | 3.5 FTE facilitators +  IDOCC project manager, project coordinator, research associate; remainder of HR provided by the practice as part of day-to-day work | Champlain  LHIN area;  Ottawa | MOHLTC $4  million;  Champlain LHIN; and sponsored in part by Pfizer Canada Inc. |
| 9. IMPACT | 4.0 FTE research staff;  3.5 FTE Pharmacists | Hamilton  (McMaster);  Ottawa  (EBRI);  Vancouver  (UBC) | $2.5 million  PHCTF 2004-6; MOHLTC now as program |
| 10. IMPROVE | Not identified | London | MOHLTC  Enhancing  Quality in  Primary Health  Care Program;  ICES support |
| 11. Improvements in Pain  Management Project | 0.1 FTE project design staff; most work absorbed | Hamilton | Dept of Family medicine |
| **Project** | **FTE & Role** | **Location** | **Funder** |
|  | by existing staff (2 physicians, 1 pharmacists, 1 OT, 1 social worker); 1.0  FTE existing Occupational Therapist specializing in pain management |  | $100,000 |
| 12. Partnership for Health,  Southwest LHIN | Not clearly identified; involves program staff, research staff | London;  Southwest  LHIN | Ministry of Finance in partnership with  MOHLTC |
| 13. Primary and Community  Care Committee (PCCC) | Not identified | Not identified | MOHLTC & OMA |
| 14. Quality Improvement  Strategic Pillar: University of  Toronto School of Family  Medicine | Absorbed internally in regular staffing | Toronto | U of T |
| 15. Quality Indicator Project | Not specifically identified other than research assistants | Kingston  (Queen’s  University) | MOHLTC |
| 16. *Quality* in Family Practice  Project | 1.0 FTE admin assistant since 2006;  1.0 FTE Quality Project planning Coordinator since  2008;  Main research supports  provided through faculty  FTEs | Hamilton (McMaster) | Originally was  $250,000 MOHLTC  funding (through  PHCTF); Now $500,000 shared between  MOHTC &  OCFP |
| 17. Resident First Initiative  Ontario Health Quality Council (OHQC) work with long term care | 15 FTEs QI facilitators | Throughout province | Not provided |
| 18. Violence Reduction  Project | Not identified (2010 start) | Not identified | MOHLTC  Workplace  Safety envelope $250,000 + inkind from OCFP |
| 19. The Change Foundation  Projects | Not identified (projectspecific) | Not identified | Not identified |
| 20. Using Computerized  Decision Support in Primary  Care | Not identified; varied depending on phase | virtual | Multiple  Funders: Health  Canada, Ontario MOHLTC, CIHI; peer-reviewed grant support for each phase |
| 21. Web-based Patient Self-Management | 2.0 FTEs to run project;  0.2 FTE for system support | virtual | MOHLTC  $200,000 under  Enhancing  Quality in PHC  (EQPHC) |
